# Supplementary material for: Associations between hematologic dynamics during pregnancy and obstetric complications: A retrospective observational study
Source: PLoS Med. 2026 May 20;23(5):e1004747. doi: 10.1371/journal.pmed.1004747 (PMC13215607; doi:10.1371/journal.pmed.1004747)
Supplement: S1 Appendix — Text A. List of hematology-related problems used for exclusion. Text B. Detail on instruments used to measure Complete Blood Counts. Text C. Detail on inclusion criteria for routine pre-pregnancy CBCs. Text D. Estimation of body mass index (BMI) in discovery and validation cohorts. Text E. Mathematical formulation of mixed-effect model for longitudinal dynamics. Text F. Calculation of biological variation. Text G. Definition of hypertensive disorders of pregnancy. Text H. Definition of small for gestational age. Text I. Mathematical formulation for association analysis with adverse outcomes. Text J. Rare dynamic definition. Text K. List of phecodes used to detect pre-existing and novel autoimmune conditions and perinatal infections. Text L. List of medications not likely to affect complete blood counts used to define subset for sensitivity analysis. Fig A. 95% intervals of complete blood count (CBC) indices of interest on the Siemens Advia 2120, Sysmex XE-5000 instruments, and Sysmex XN-9000 instruments. Discrepancies between machines were so great for MPV that it was subsequently excluded from all further analysis. Abbreviations: HCT,hematocrit; HGB, hemoglobin; WBC, white cell count; RBC, red cell count; PLT, platelet count; MCV, mean corpuscular volume; MCH, mean corpuscular hemoglobin; MCHC, mean corpuscular hemoglobin concentration; MPV, mean platelet volume. Fig B. Frequency of available CBCs by gestational week in discovery cohort pregnancies. Shaded red areas indicate chosen windows for which we considered CBCs in gestation (7–14 weeks and 26–29 weeks). The pre-delivery time point is not shaded as it is individualized and fell between 30 and 41 weeks gestation. Fig C. Sensitivity analysis of the effects of including patients with a diagnosis of anemia on reference interval determination. The figure compares gestational-age-specific intervals for all term pregnancies without complications (blue error bars), pregnancies with no diagnosis of anemia (<11 g/dL HG [file pmed.1004747.s002.docx]

**Supplementary material for**

**Associations between hematologic dynamics during pregnancy and obstetric complications: a retrospective observational study**

Table of Contents

[Supplementary Methods 2](#_Toc228253579)

[Text A. List of hematology related problems used for exclusion 2](#_Toc228253580)

[Text B. Detail on instruments used to measure Complete Blood Counts 2](#_Toc228253581)

[Text C. Detail on inclusion criteria for routine pre-pregnancy CBCs 2](#_Toc228253582)

[Text D. Estimation of body mass index (BMI) in discovery and validation cohorts 2](#_Toc228253583)

[Text E. Mathematical formulation of mixed-effect model for longitudinal dynamics 3](#_Toc228253584)

[Text F. Calculation of biological variation 3](#_Toc228253585)

[Text G. Definition of hypertensive disorders of pregnancy 4](#_Toc228253586)

[Text H. Definition of small for gestational age 5](#_Toc228253587)

[Text I. Mathematical formulation for association analysis with adverse outcomes 5](#_Toc228253588)

[Text J. Rare dynamic definition 5](#_Toc228253589)

[Supplementary figures 6](#_Toc228253590)

# **Supplementary Methods**

### Text A. List of hematology related problems used for exclusion

Patients diagnosed with any of the following blood disorders prior to pregnancy were excluded from the analysis in the discovery cohort. Diagnoses were ascertained from a clinical problem list in the obstetric record. Diagnostic exclusion criteria included:

- Immune/idopathic thrombocytopenia purpura (ITP)
- Alpha thalassemia trait and Hemoglobin H disease
- Beta thalassemia trait, beta thalassemia intermedia, and beta thalassemia major
- Sickle cell disease, including sickle-beta-zero thalassemia, sickle-beta-plus thalassemia, and hemoglobin SC disease (Subjects with sickle trait were included in the analysis)
- Hemoglobin E heterozygosity or homozygosity
- G6PD deficiency homozygosity (but not G6PD carriers)
- Hereditary spherocytosis
- Hereditary elliptocytosis
- Hereditary xerocytosis
- Pyruvate kinase deficiency
- Hemolytic anemia

In the validation cohort, patients with blood disorders assigned the following ICD 10 codes were excluded: D66 D67 D68 D69.

## Text B. Detail on instruments used to measure Complete Blood Counts

CBCs were measured on Siemens Advia 2120 instruments until 2012 and on Sysmex XE-5000 instruments until 2016. Most CBC measurements performed between 2016 and 2024 were made on Sysmex XN-9000 instruments. All CBCs were measured in clinical laboratories that participated in proficiency testing programs.

## Text C. Detail on inclusion criteria for routine pre-pregnancy CBCs

Routine pre-pregnancy CBCs were considered if the individual had no more than two complete blood count (CBC) tests in their medical record within the five years before their pregnancy separated by at least 6 months, and had no encounter marked with an ICD9 code related to pregnancy (V20-V29, 630-679), cancer (140-239), or infection (1-139) between 180 days prior the CBC and until the day of the pregnancy in consideration. A histogram of the PheCodes [1] for the included patients is available in **Fig G**.

## Text D. Estimation of body mass index (BMI) in discovery and validation cohorts

​For each pregnancy included in the discovery and validation cohort, we standardized prenatal weight/BMI to values at 12 weeks’ gestation, interpolating or extrapolating if necessary, according to the following procedure in order of priority, as described previously in [26]:

1. If there is a measurement at exactly 12 weeks’ gestation, use that measurement. (If there are multiple measurements at 12 weeks’ gestation, take the mean.)
2. If there is at least one measurement prior to 12 weeks’ gestation and at least one between 12 and 20 weeks’ gestation, calculate the 12-week measurement using the following procedure. If there are at least 5 prenatal measurements total for that pregnancy, fit a curve using cubic splines to model the relationship between gestational age and the measure. If there are fewer than 5 measurements, use linear regression to fit that curve instead. Predict the 12-week measurement based on the individual-specific fitted curve (i.e., interpolation).
3. If there is at least one prenatal measurement but not enough to perform procedure #2, fit a linear mixed-effects model, modeling the relationship between gestational age and the measure using cubic splines, using prenatal data from all pregnancies in the cohort. Use random intercepts to account for multiple prenatal measurements associated with the same pregnancy. (This population-level curve is estimated only once for the entire population.) Predict the 12-week measurement using the population-averaged curve (i.e., extrapolation).
4. If a pregnancy has no prenatal measurements, then the prenatal measurement cannot be standardized to 12 weeks and is treated as missing.

## Text E. Mathematical formulation of mixed-effect model for longitudinal dynamics

Intra-patient trends in CBC indices over the course of prenatal visits were analyzed using a linear mixed-effects model adjusted for age and BMI at 12 weeks’ gestation. Random effects were added for the individual pregnancy to account for physiological variation at pre-delivery, for individual patients, for year of delivery, for parity, and for different prenatal sites of care to account for machine calibration biases. If we consider the example of RBC dynamics between a [7,14] week visit and a [26,29] week visit, then the model is formally written as:

$${RBC}_{i}=\beta_{0}+ {\beta_{1}Visit}_{i}+ {\beta_{2}BMI}_{i}+ {\beta_{3}Age}_{i}+b_{0i}+ b_{0j}+b_{0k}+b_{0y}+b_{0z}$$

where $i$ denotes the pregnancy, $j$ denotes the prenatal care location, $k$ denotes the year, $y$ denotes parity, and $z$ denotes the individual. ${\beta_{1}Visit}_{i}$ is the contribution that the distance between two successive prenatal visits, or pre-pregnancy to the first prenatal visit, contributes to the change in that marker.

## Text F. Calculation of biological variation

Biological variation is used as a benchmark for changes in CBC results. Intra-person biological variation is estimated for each index by the European Federation of Clinical Chemistry and Laboratory Medicine (EFLM) Working Group on Biological Variation using meta-analyses of high quality studies, ascertained through the Biological Variation Data Critical Appraisal Checklist, to describe the variability of laboratory indices within individuals. The biological variation was provided as a coefficient of variation which was used to calculate values representing 2 standard deviations from the mean of each CBC index in our discovery cohort. The column “Biological variation” is the intra-patient coefficient of variation for that marker as available from [31] at the link <https://biologicalvariation.eu>. The column “Mean” is the value used to estimate the standard deviation and is calculated as the mean at the pre-pregnancy point across patients with no adverse events. The column “2STD cutoff” is two times the biological variation multiplied by the mean of the pre-pregnancy timepoint, and serves as the cutoff to which we compare subsequent dynamics of the CBC indices (see **Figure 3 and Table 2**).

| **CBC index** | **Biological variation (%)** | **Mean** | **2STD cutoff** |
| --- | --- | --- | --- |
| Hematocrit (%) | 2.8 | 39 | 2.2 |
| Hemoglobin | 1.6 | 13.2 | 0.4 |
| White blood cell count | 10.8 | 7.3 | 1.6 |
| Red blood cell count | 2.6 | 4.4 | 0.2 |
| Platelet count | 7.5 | 271 | 40.7 |
| Mean Corpuscular Volume | 0.8 | 88 | 1.4 |
| Mean Corpuscular Hemoglobin | 0.9 | 30 | 0.5 |
| Red blood cell Distribution Width (%) | 1.6 | 12.8 | 0.4 |
| Mean Corpuscular Hemoglobin Concentration | 1 | 34.2 | 0.7 |

### Text G. Definition of hypertensive disorders of pregnancy

Pregnancies in the discovery cohort were considered affected by a hypertensive disorder of pregnancy (HDP), including gestational hypertension or preeclampsia, if there was no evidence of pre-existing chronic hypertension and at least one of the following was found in the medical record:

- Two or more systolic blood pressure (BP) readings >= 140 mmHG and/or diastolic BP readings >=90 mmHG greater than 4 hours apart after 20 weeks of gestation and up to one week postpartum;
  OR
- Recording of HDP as an indication for induction, indication for cesarean or complication of labor in the labor and delivery record;

OR

- One elevated systolic BP >=140 mmHG and/or diastolic >=90 mmHG after 20 weeks of gestation with evidence of preeclampsia (see below).

Pregnancies in the discovery cohort were considered to be affected by preeclampsia if there was:

- Hypertensive disorder of pregnancy (as above)

AND

- Evidence of preeclampsia as defined by one or more of the following:
  - 24 hour timed urine protein >=300 mg/day
    OR
  - Urine protein to creatinine ratio>=0.3 mg/mg Cr
    OR
  - Serum creatinine >=1.1 mg/dL
    OR
  - Platelets < 100 10^9^/L
    OR
  - AST>40 U/L
    OR
  - ALT>40 U/L
    OR
  - Spot/dipstick urine protein reading >=2+ in the absence of timed urine or urine protein to creatinine ratio
    OR
  - ICD code for eclampsia

These HDP definitions were validated via blinded chart review by maternal fetal medicine specialists with an accuracy of 90%.

In the validation cohort, pregnancies were considered affected by HDP if there was no evidence of pre-existing chronic hypertension and at least one of the following was found in the medical record:

- SBP ≥ 140 or DBP ≥ 90 on 2+ occasions which are at least 4 hours apart – 20 weeks gestational age to 7 days postpartum
- Recording of an HDP/Preeclampsia delivery ICD code

Differences between the discovery and validation definitions are a product of differences in coding practices when MGB switched to Epic in 2016.

### Text H. Definition of small for gestational age

Newborns were considered small for gestational age (SGA) if their birthweight was less than the 10^th^ percentile of babies of the same sex delivered at the same gestational week. See table below for the 10th percentile weight cutoffs used for the analyzed cohort defined in [37].

|  |  | **Gestational age at delivery (weeks)** | | | | | | | | | | | |
| --- | --- | --- | --- | --- | --- | --- | --- | --- | --- | --- | --- | --- | --- |
|  |  | **30** | **31** | **32** | **33** | **34** | **35** | **36** | **37** | **38** | **39** | **40** | **41** |
| **Weight (grams)** | **Female** | 965 | 1180 | 1390 | 1638 | 1872 | 2099 | 2299 | 2495 | 2694 | 2834 | 2919 | 2949 |
|  | **Male** | 1044 | 1241 | 1475 | 1712 | 1957 | 2192 | 2410 | 2609 | 2807 | 2947 | 3029 | 3063 |

## Text I. Mathematical formulation for association analysis with adverse outcomes

Odds ratios were obtained using generalized estimating equations (GEE) for logistic regression adjusted for BMI at 12 weeks’ gestation, age of the individual, insurance, parity, and race/ethnicity. Clustering was at the level of the individual and an exchangeable correlation structure was used. The model for the mean is as follows:

$$logit\left( E[Y_{i}] \right)=\beta_{0}+ \beta_{1}Indicator_{i}+ {\beta_{2}BMI}_{i}+ {\beta_{3}Age}_{i}+\beta_{4}{Insurance}_{i}+\beta_{5}{Parity}_{i}+\beta_{6}{Race/ethnicity}_{i}$$

where $E[Y_{i}]$ denotes the expected value, or probability, of an adverse outcome of interest (small for gestational age, preeclampsia, hypertensive disorder of pregnancy, preterm delivery, transfusion at or after delivery) or a composite of HDP, preterm delivery, and SGA, and $i$ denotes the pregnancy. $Insurance$ and $Race/ethnicity$ categories are presented in **Table 1**. Race/ethnicity is included in this analysis to capture disparities in adverse pregnancy outcomes among self-reported racial groups and not to imply any biological mechanism underlying these disparities. $Indicator$ is a binary indicator variable marking whether the individual fell within or outside of this study’s reference intervals or those in the literature, or met definition of a rare dynamic (see Rare dynamic definition) depending on the test at hand. For reference interval tests, we considered whether CBC values in a pregnancy fell outside of the reference interval for that index at 26-29 weeks, in a one-sided (above or below) and two-sided (above and below) fashion. Rare behavior tests are described in Rare dynamic definition. The package geepack in R was used to estimate regression coefficients [32, 33, 34]. P-values were corrected with a Bonferroni correction, the level of which was test specific and is reported in appropriate figure and table captions (**Figure 4**, **Table 2** and **Tables S6-8**). For this analysis we excluded pregnancies with a HDP diagnosis before 29 weeks as determined by an elevated blood pressure.

## Text J. Rare dynamic definition

We analyzed data from three time intervals (pre-pregnancy to 7-14 weeks’ gestation, 7-14 weeks’ to 26-29 weeks’ gestation, and 26-29 weeks’ gestation to pre-delivery). In the main analysis we focused on the time interval between 7-14 weeks and 26-29 as the most unbiased and practical time point that for detection of pregnancies with adverse outcomes before delivery. As CBCs among young people capable of pregnancy are not routinely indicated as screening tests outside of pregnancy, individuals with pre-pregnancy CBCs may have selection bias and may not reflect indices in the general population. Additionally, 64% of analyzed pregnancies have a CBC at both 7-14 weeks’ and 26-29 weeks’ gestation, compared to just 9% and 10% respectively for a CBC at both pre-pregnancy and 7-14 weeks’. Analyzing changes between 26-29 weeks’ gestation and pre-delivery would not allow for timely prediction of complications. Thus, we focused on the interval between 7-14 weeks’ and 26-29 weeks’ gestation for prediction of adverse outcomes.

For this interval between 7-14 weeks’ and 26-29 weeks’ gestation we compute the change in the CBC indices (delta) for each pregnancy. We considered a delta to be stable if the absolute value of the change is less than 2*x* the biological variation for that index, increasing if it is greater than 2*x* the biological variation, and decreasing if it is less -2*x* of the biological variation. Biological variation for each marker was derived from the EFLM Biological Variation Database [31]. We reported the estimated standard deviations above in Calculation of biological variation. The prevalence of observed CBC index dynamics was calculated from the percentage of patients with positive, non-changing, or negative deltas. The directionality of the rare behavior was decided based on the least likely of the three types of change between 7-14 and 26-29 weeks’ gestation (see **Table S6**). After directionality was chosen, we tested 100 thresholds linearly spaced between minimum and maximum delta values for each index, and selected the thresholds yielding the highest positive predictive value (PPV) and a significant odds ratio (OR). We focused on PPV because it helps assess a marker’s potential clinical significance, which typically requires that the PPV significantly exceed the prevalence of the outcome being predicted. Significance of the OR was decided by comparing p-values with a Bonferroni corrected threshold. See **Table 2** in main text for rare dynamics significantly associated with adverse outcomes, and **Table S14** for a summary of other adverse outcomes and indices.

Text K. List of Phecodes used to detect pre-existing and novel autoimmune conditions and perinatal infections.
For autoimmune conditions, we considered all Phecodes in the categories “Other Immune Disorders” and “Autoimmune arthritis and psoriasis”, which included the Phecodes 279.[00|11|10|20|70|80], 714.[00|10|20] and 715.[00|10|20|30] for a total of 48 corresponding ICD-9 codes.

For infections, we considered any Phecode in the categories “Intestinal infection”, “bacterial infection”, “viral infection”, “Sexually transmitted infections (not hiv or hepatitis)”, “Postoperative infection”, “Infectious diseases”, which corresponded to all Phecodes with a number < 100, for a total of 2,017 ICD-9 codes.

We used Phecodes map 1.2[2].

Text L. List of medications not likely to affect complete blood counts used to define subset for sensitivity analysis.
Multivitamins, Prenatal vitamins, Influenza vaccine, Ibuprofen, Acetaminophen, Docusate sodium, D Globin Immune rho, Albuterol, Folic acid, Fioricet, Lorazepam, Fluticasone, Ergocalciferol, Vitamin D.

# **Supplementary figures**


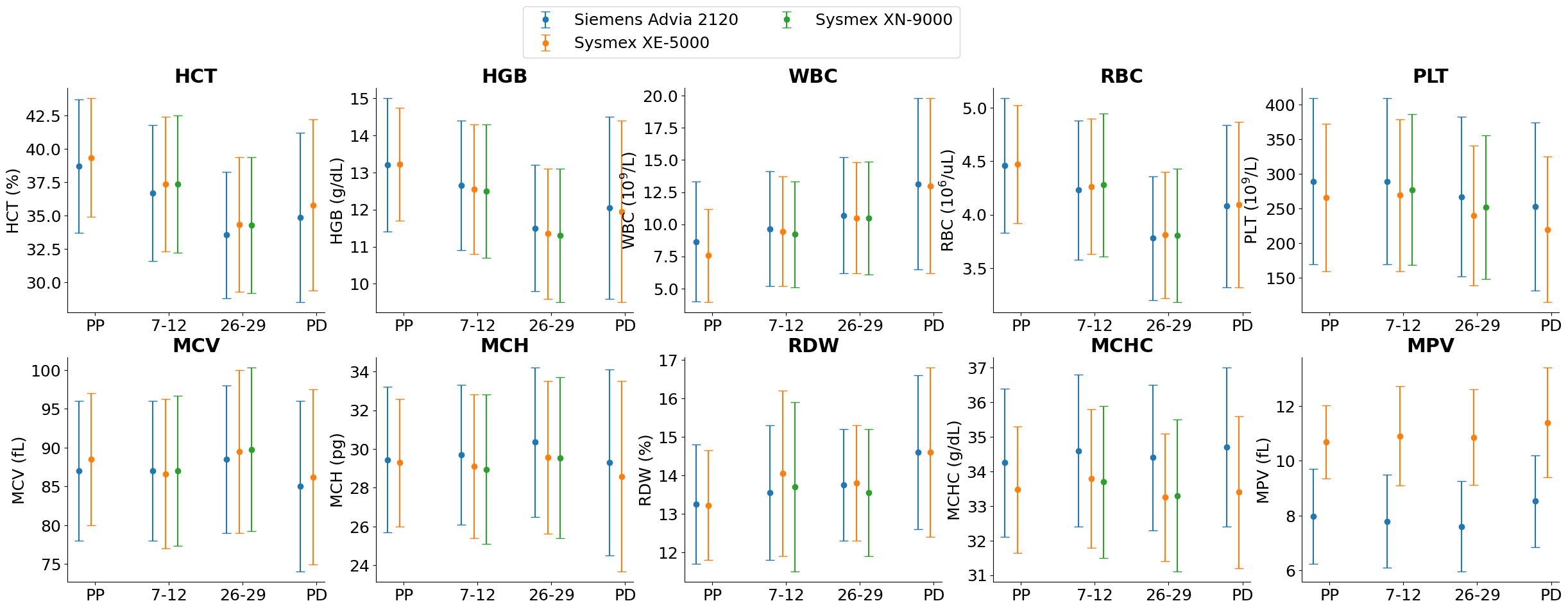


**Fig A.** 95% intervals of complete blood count (CBC) indices of interest on the Siemens Advia 2120, Sysmex XE-5000 instruments, and Sysmex XN-9000 instruments. Discrepancies between machines were so great for MPV that it was subsequently excluded from all further analysis*. Abbreviations*: HCT – Hematocrit, HGB – Hemoglobin, WBC – White cell count; RBC – Red cell count; PLT – Platelet count; MCV – Mean corpuscular volume; MCH – Mean corpuscular hemoglobin; MCHC - Mean corpuscular hemoglobin concentration; MPV – Mean platelet volume


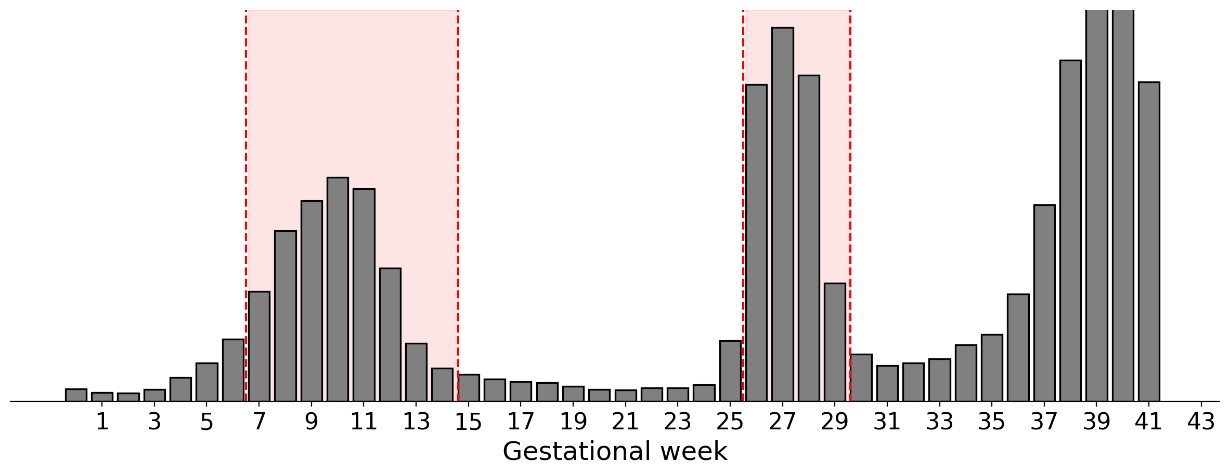


**Fig B**. Frequency of available CBCs by gestational week in discovery cohort pregnancies. Shaded red areas indicate chosen windows for which we considered CBCs in gestation (7-14 weeks and 26-29 weeks). The pre-delivery timepoint is not shaded as it is individualized and fell between 29 to 41 weeks gestation.


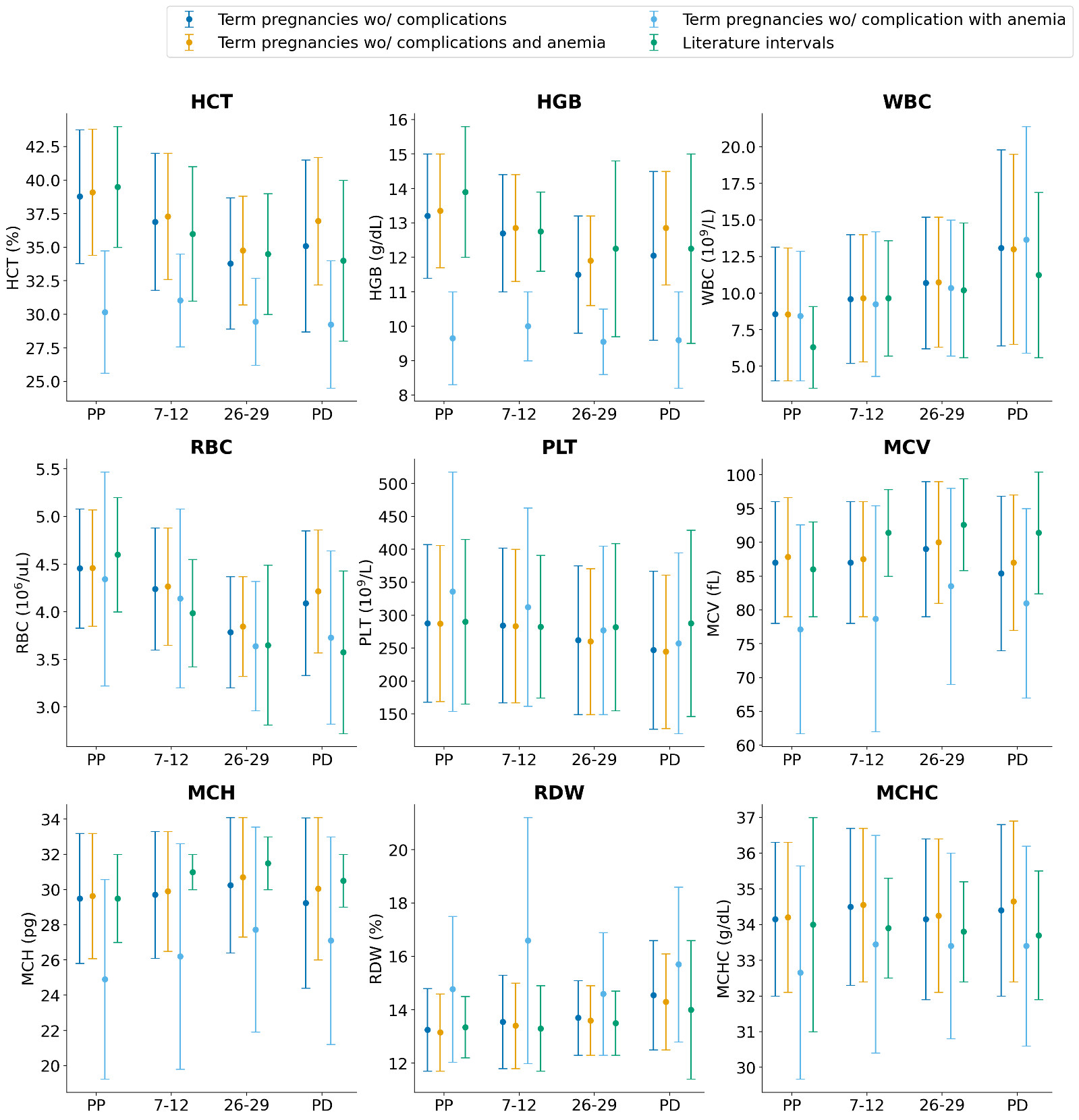


**Fig C***.* **Sensitivity analysis of the effects of including patients with a diagnosis of anemia on reference interval determination**. The figure compares gestational-age-specific intervals for all term pregnancies without complications (dark blue error bars), pregnancies with no diagnosis of anemia (<11 g/dL HGB at PP, 7-14 and PD, and <10.5 g/dL at 26-29 weeks, orange error bars), pregnancies considered anemic (light blue error bars), and literature trimester-specific intervals (green error bars), all in the discovery cohort. Up to 15% of pregnancies met criteria for anemia at each timepoint. *Abbreviations*: HCT – Hematocrit, HGB – Hemoglobin, WBC – White cell count; RBC – Red cell count; PLT – Platelet count; MCV – Mean corpuscular volume; MCH – Mean corpuscular hemoglobin; MCHC - Mean corpuscular hemoglobin concentration; MPV – Mean platelet volume


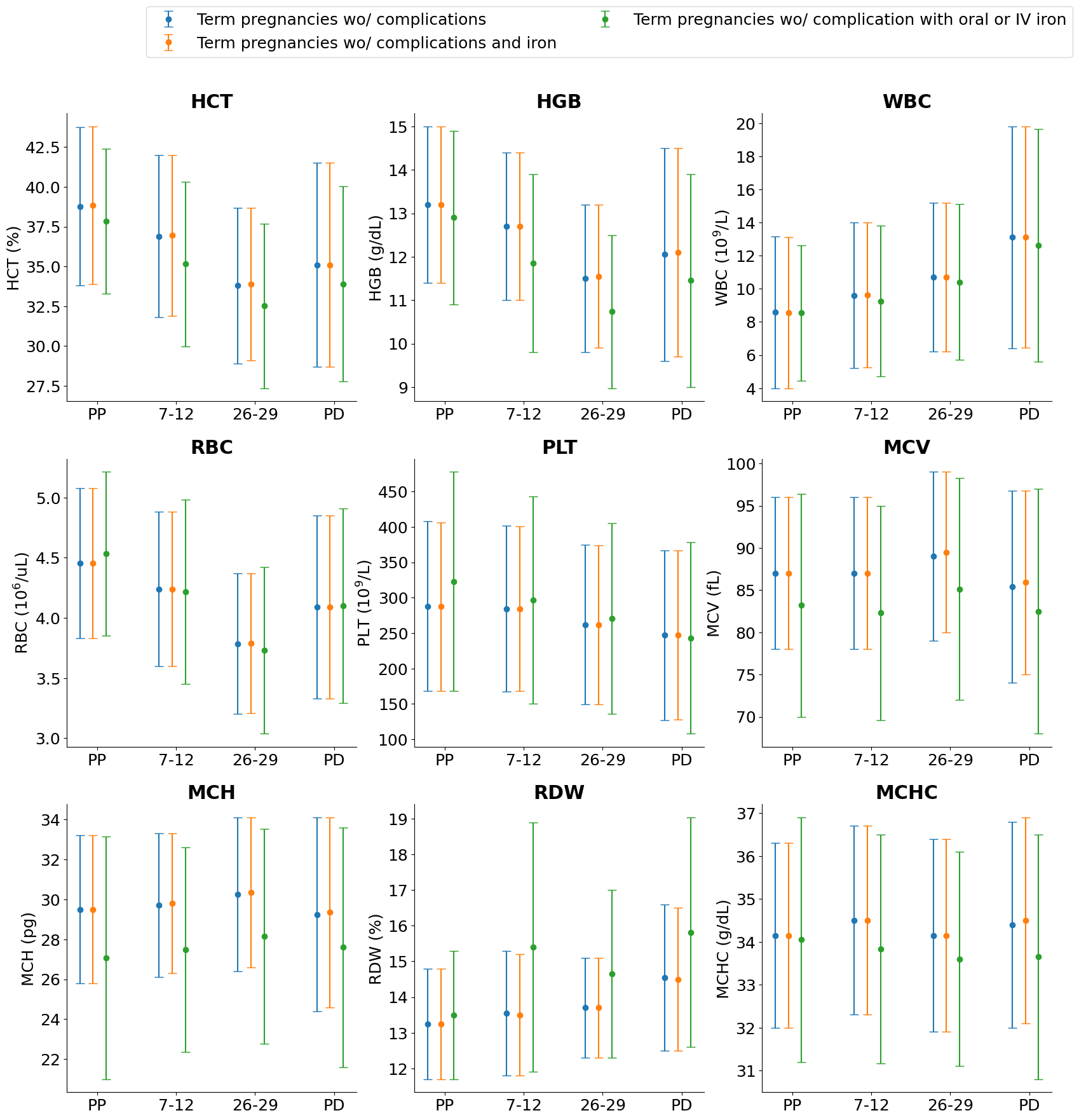


**Fig D**. **Sensitivity analysis of the effects of including patients with recorded iron supplementation on reference interval determination**. The figure compares gestational-age-specific intervals for all term pregnancies without complications (blue error bars), pregnancies with no record of either oral or IV iron supplementation in their medical record (orange bars), and pregnancies with recorded oral or IV iron supplementation (green error bars, all in the discovery cohort). N =1503 of 37,709 term pregnancies without complications had recorded IV or oral iron in their medical record. *Abbreviations*: HCT – Hematocrit, HGB – Hemoglobin, WBC – White cell count; RBC – Red cell count; PLT – Platelet count; MCV – Mean corpuscular volume; MCH – Mean corpuscular hemoglobin; MCHC - Mean corpuscular hemoglobin concentration; MPV – Mean platelet volume


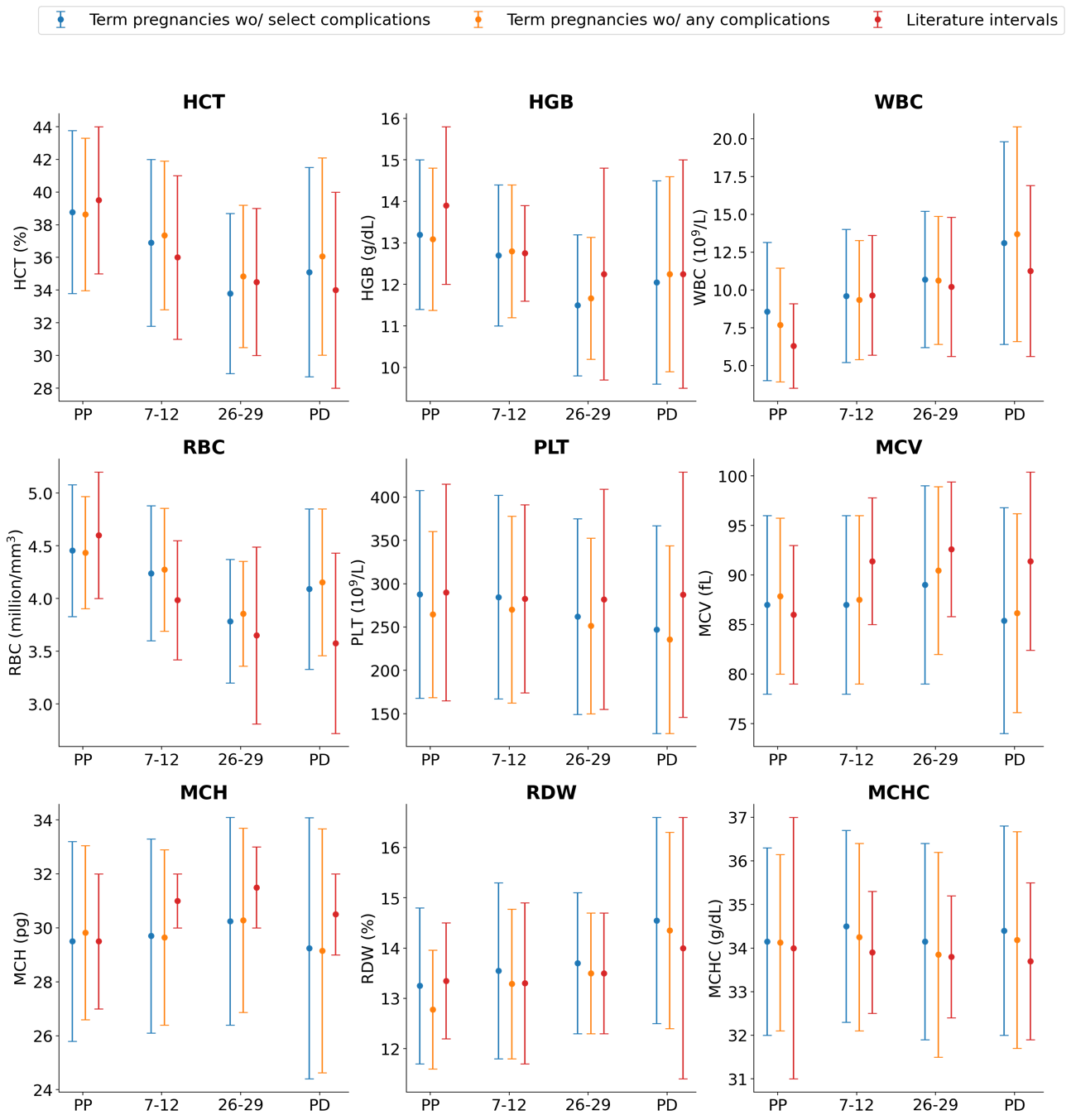


**Fig E**. **Sensitivity analysis of the effects of including patients with chronic or pregnancy-related conditions on reference intervals**. Comparison of gestational-age-specific intervals for term pregnancies without complications in discovery, pregnancies in individuals without chronic or pregnancy-related conditions (N=1460) in discovery and literature trimester-specific intervals. *Abbreviations*: HCT – Hematocrit, HGB – Hemoglobin, WBC – White cell count; RBC – Red cell count; PLT – Platelet count; MCV – Mean corpuscular volume; MCH – Mean corpuscular hemoglobin; MCHC - Mean corpuscular hemoglobin concentration; MPV – Mean platelet volume

**
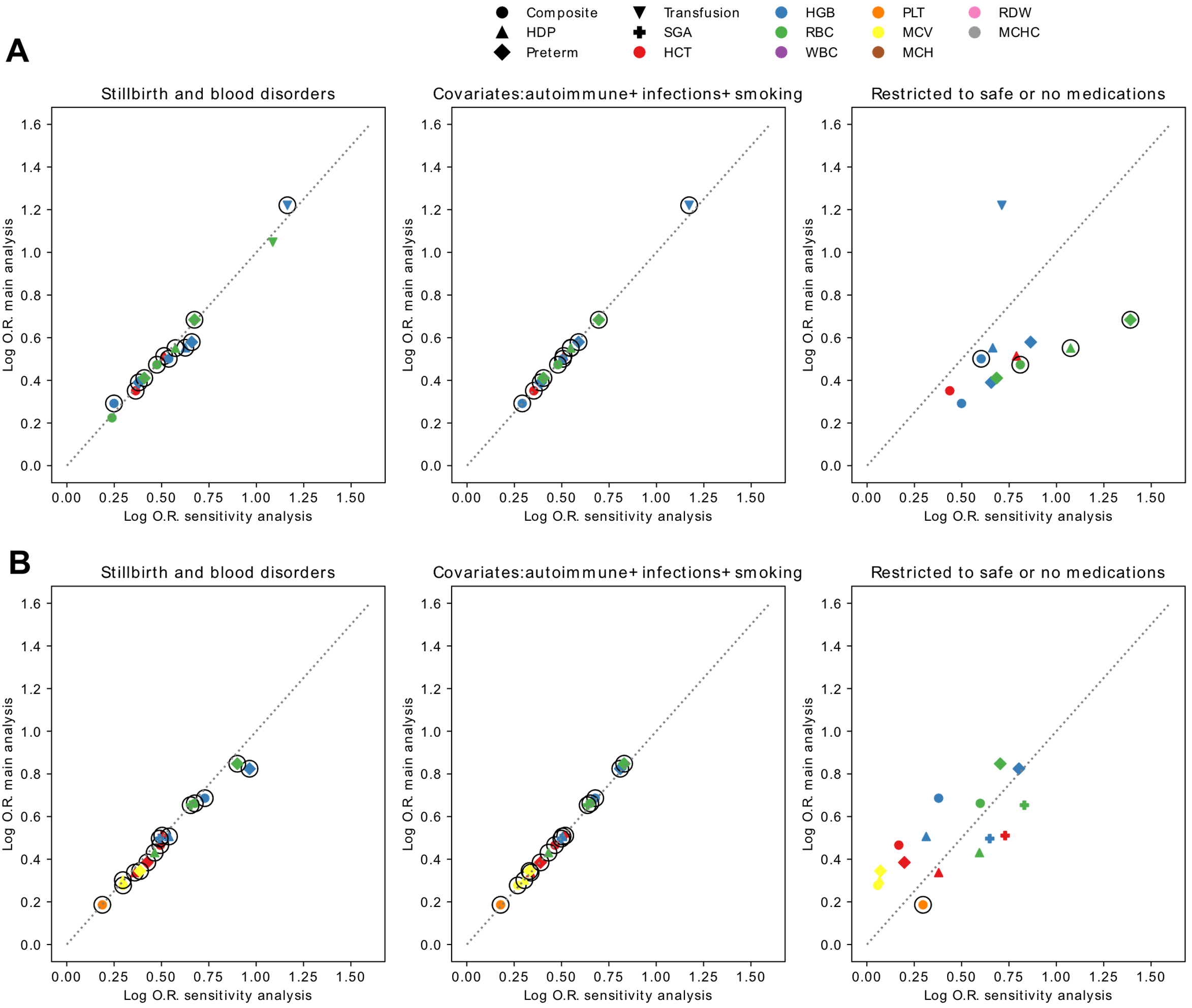
**

**Fig F.** Odds ratio comparison between main analysis and sensitivity analyses for out of range associations (panel A) and rare dynamics (panel B). Circled in black those associations that were significant in both main and sensitivity analysis. The hemoglobin (HGB) outlier can be attributed to a larger confidence interval [1.49, 26.49] that no longer meets Bonferroni correction, but is still nominally significant. Abbreviations: HCT – Hematocrit, HGB – Hemoglobin, WBC – White cell count; RBC – Red cell count; PLT – Platelet count; MCV – Mean corpuscular volume; MCH – Mean corpuscular hemoglobin; MCHC - Mean corpuscular hemoglobin concentration; MPV – Mean platelet volume; HDP – Hypertensive Disorder of pregnancy; SGA – small for gestational age; O.R. – Odds ratio

**Fig G.** Histogram of 50 most frequent PheCodes for visits corresponding to the pre-pregnancy CBCs considered. Considered pre-pregnancy CBCs are described in Text S3.

**References**

1. Wu P, Gifford A, Meng X, Li X, Campbell H, Varley T, et al. Mapping ICD-10 and ICD-10-CM Codes to Phecodes: Workflow Development and Initial Evaluation. JMIR Med Inform. 2019;7: e14325. doi:10.2196/14325

2. Denny JC, Bastarache L, Ritchie MD, Carroll RJ, Zink R, Mosley JD, et al. Systematic comparison of phenome-wide association study of electronic medical record data and genome-wide association study data. Nat Biotechnol. 2013;31: 1102–1110. doi:10.1038/nbt.2749
