# Supplementary material for: Associations between hematologic dynamics during pregnancy and obstetric complications: A retrospective observational study
Source: PLoS Med. 2026 May 20;23(5):e1004747. doi: 10.1371/journal.pmed.1004747 (PMC13215607; doi:10.1371/journal.pmed.1004747)
Supplement: Attachment — Submitted filename: response_to_reviwers.docx [file pmed.1004747.s024.docx]

February 7th, 2026

Dear Dr. Bruijn,

Thank you for considering the revision of our manuscript entitled “Associations between hematologic dynamics during pregnancy and obstetric complications” for publication in *PLOS Medicine.* We appreciate the thoughtful review comments which have helped us strengthen the manuscript.

We have provided a detailed, point-by-point response and description of the corresponding manuscript revisions in the following text.

**Associate Editor**

*We ask you to address all concerns, but specifically the concerns regarding remaining confounding, and risk of selection bias.*

**Reply:** We provide detailed responses to address these important concerns below, describing our approach to minimizing potential confounding in response to Reviewer 2’s 3rd comment, and our approach to minimizing risk of selection bias in response to the Academic Editor’s comment directly below.

**Academic Editor**

*(E.1) There seems to be a high risk of selection bias, which is maximized by including only patients who delivered after 30 weeks, and by only including live births.*

**Reply:** We appreciate the editor’s concern regarding potential selection bias. The inclusion criterion requiring delivery after 29 weeks was driven by the study's objectives. Our first main objective was to provide CBC reference intervals for uncomplicated pregnancies in individuals without blood disorders, and standard practice for reference interval determination involves excluding those with complications, including delivery before 29 weeks. Our other main objective was to evaluate associations between CBC measurements made at 26-29 weeks’ gestation (the exposure) and subsequent adverse pregnancy outcomes. Including only patients who delivered after 29 weeks is necessary to avoid analyzing individuals for whom the outcome occurred before the exposure. The findings related to associations between rare CBC dynamics and out-of-range CBC indices at 26-29 weeks’ gestation apply only to pregnancies that are uncomplicated as of 29 weeks’ gestation. In contrast, the CBC reference intervals for uncomplicated pregnancies we report provide physiologic context that we believe is relevant for all pregnancies.

To evaluate the possibility that selection bias was introduced by other inclusion criteria including the live birth requirement, we conducted a sensitivity analysis in which we expanded the discovery cohort to include pregnancies that delivered after 29 weeks but had been excluded due to stillbirths or a blood disorder. These exclusions are the only ones that do not directly interfere with outcome or exposure ascertainment. In this sensitivity analysis, stillbirths were added to the composite outcome, and the presence of a blood disorder was added to our GEE model as a covariate. Odds ratios were consistent with, and in many cases higher than, those from the primary analysis (see figure below).

Also, to emphasize that our study’s target population is pregnancies ongoing at 29 weeks with a 26-29 week CBC and that our results may not generalize to pregnancies outside that target populations, we include demographic comparisons between included and excluded pregnancies. We first note that we find no evidence for demographic differences with the exception of parity between uncomplicated and complicated pregnancies in our target study population (**Table 1**), supporting the validity of our findings for the target population. In our revised manuscript, we also compare demographic characteristics between pregnancies in our target population and those outside our target population: those excluded due to stillbirth or delivery <29 weeks, and all excluded pregnancies in both discovery and validation. We observed differences across groups, including a higher proportion of pregnancies with public (vs. private) insurance, a higher proportion of individuals who self-identified as Black or Hispanic, and a higher BMI at 12 weeks’ gestation in the excluded cohorts compared with the included. These differences may reflect broader sociodemographic factors associated with increased risks of stillbirth and preterm delivery in lower socioeconomic strata [Spong et al, 2011], as well as reduced access to prenatal care, which may have contributed to exclusion due to missing CBC data. While these differences between our target population and those outside our target population are clinically and socially meaningful and are now reported in the manuscript, they do not affect the validity of our results for pregnancies ongoing at 29 weeks with 26-29 week CBCs.

These results are summarized in the *Supplementary Material* and referenced in the *Results*. The *Discussion* is further expanded to address this limitation.

Spong et al. Disparities in Perinatal Medicine: Preterm Birth, Stillbirth, and Infant Mortality. Obstetrics & Gynecology 117(4):p 948-955, April 2011. | DOI: 10.1097/AOG.0b013e318211726f

**Results**

**Page 7, Lines 297-302**

We analyzed the demographic characteristics of pregnancies that were excluded from the analysis (**Table S2**), which show a higher number of pregnancies with public insurance, a higher number of individuals who self-identified as Black or Hispanic, and a higher BMI. Their exclusion could be due in part to socio-economic factors that led to reduced access to care and prevented us from collecting necessary CBC data for the analysis.

**Page 8, Lines 373 - 377**

Sensitivity analysis in the discovery cohort including pregnancies resulting in stillbirth or reporting blood disorders (**Table S10**), adding autoimmune conditions, infections, and smoking status (**Table S11**), or restricting to those pregnancies that did not report medications possibly affecting CBC dynamics (**Table S12**), showed consistent results with the primary analysis (**Figure S7)**.

**Page 10, Lines 425-430**

We evaluated the robustness of the thresholds identified in the discovery cohort through three sensitivity analyses. These included: (1) expanding the cohort to include pregnancies complicated by stillbirth or blood disorders (**Table S15**); (2) additionally adjusting for autoimmune conditions, infections, and smoking status (**Table S16**); and (3) restricting the analysis to pregnancies not exposed to medications that could plausibly affect CBC dynamics (**Table S17**). Across all analyses, results were consistent with the primary findings (**Figure S7**).

**Discussion**

**Page 12, Lines 520-523**

Second, our findings regarding CBC-associated risk considered only uncomplicated pregnancies or those that developed complications after 29 weeks’ gestation, and therefore these results may not generalize beyond this target population to pregnancies with complications occurring prior to 29 weeks.

**Figure S7** Odds ratio comparison between main analysis and sensitivity analyses for out of range associations (panel A) and rare dynamics (panel B). Circled in black those associations that were significant in both main and sensitivity analysis. Depicted odds ratios are limited to those previously found significant in the main analysis. The hemoglobin (HGB) outlier can be attributed to a larger confidence interval [1.49, 26.49] that no longer meets Bonferroni correction, but is still nominally significant. *Abbreviations*: HCT – Hematocrit, HGB – Hemoglobin, WBC – White cell count; RBC – Red cell count; PLT – Platelet count; MCV – Mean Corpuscular Volume; MCH – Mean corpuscular hemoglobin; MCHC - Mean corpuscular hemoglobin concentration; MPV – Mean platelet volume; HDP – Hypertensive Disorder of pregnancy; SGA – small for gestational age; O.R. – Odds ratio

**Table S2.** Comparison of demographics included/excluded pregnancies during the development of our discovery and validation cohorts.

| Variable | Included (N=45,992) | Excluded due to stillbirth & early delivery (N=283) | Excluded (N=8,086) | Included (N=48,868) | Excluded due to stillbirth & early delivery (N=1,035) | Excluded (N=80,831) |
| --- | --- | --- | --- | --- | --- | --- |
| **Age** | 32.0 (6) | 31.0 (7) | 30.0 (7) | 33.0 (5) | 32.0 (6) | 33.0 (5) |
| **Insurance** |  |  |  |  |  |  |
| Public | 11632 (25) | 100 (35) | 2788 (34) | 6090 (12) | 240 (23) | 16816 (21) |
| Private | 30322 (66) | 152 (54) | 4033 (50) | 42335 (87) | 783 (76) | 62905 (78) |
| None/limited | 4038 (9) | 31 (11) | 1265 (16) | 443 (1) | 12 (1) | 1110 (1) |
| **Race/Ethnicity** |  |  |  |  |  |  |
| Asian | 4085 (9) | 25 (9) | 747 (9) | 5557 (11) | 88 (9) | 8362 (10) |
| Black | 3001 (7) | 35 (12) | 1012 (13) | 3052 (6) | 186 (18) | 8504 (11) |
| Hispanic or latino | 5621 (12) | 48 (17) | 1164 (14) | 4097 (8) | 99 (10) | 8593 (11) |
| White | 27627 (60) | 129 (46) | 4021 (50) | 34751 (71) | 609 (59) | 52105 (64) |
| Other | 4229 (9) | 38 (13) | 780 (10) | 38 (0) | 2 (0) | 121 (0) |
| Unknown | 1040 (2) | 3 (1) | 282 (3) | 1373 (3) | 51 (5) | 3146 (4) |
| **Nulliparous** | 21525 (47) | 146 (52) | 3943 (49) | 23068 (47) | 483 (47) | 37228 (46) |
| **Gestational age at delivery** | 39.0 (2) | 30.0 (5) | 39.0 (3) | 39.0 (1) | 30.0 (7) | 39.0 (2) |
| **BMI at 12 weeks** | 25.3 (5.1) | 27.0 (6.6) | 26.6 (6.3) | 26.1 (5.5) | 28.1 (6.8) | 27.0 (6.2) |

**Reviewer #1**

*(1.1) The statistical methodology appears sound, and I acknowledge that this is not an area of my expertise.*

**Reply:** We are pleased the reviewer finds the statistical analysis in our study to be sound.

(1.2) *The purpose of the study was to evaluate the association between hematologic dynamics and pregnancy complications. The authors reported that elevated red cell measures correlated
with subsequent obstetric complications.  There were no such trend seen with platelets (which is surprising as low platelets are an identified finding in a subset of HDP--HELLP).*

**Reply:** We thank the reviewer for this comment. While HELLP syndrome is a severe form of HDP, the vast majority of pregnancies with HDP do not meet criteria for HELLP syndrome, which might explain why there was no association between low platelet counts and HDP. A prior large study [Reese at al. 2018] found that pregnancies with HDP exhibit elevated platelet counts through approximately 32 weeks of gestation. This pattern is reversed as pregnancies approach delivery, which is consistent with the observed low platelet characteristics of HELLP syndrome seen in previous findings. Our results replicate these findings. We have added a sentence to the *Discussion* to emphasize this point.

Reese, J. A. *et al.* Platelet Counts during Pregnancy. *N Engl J Med* **379**, 32–43 (2018).

**Discussion**

**Page 11, Lines 475-477**

We also found that, although not significant after multiple testing, elevated PLT count was nominally significantly associated with the composite outcomes and HDP, in line with previous findings on platelets dynamics during pregnancy[17].

(1.3) *The authors excluded patients who delivered <30w for this study, and this may have excluded those with HDP resulting in preterm delivery, and perhaps those with HELLP.*

**Reply:** Our inclusion criteria were purposefully stringent for two primary reasons. To evaluate the predictive power of these intervals for subsequent complications, we included only pregnancies in which complications developed after 29 weeks. Including cases with earlier onset would have risked incorporating CBC samples drawn after disease onset. We recognize that this approach limits the generalizability of our findings to pregnancies not complicated by early-onset HDP, delivery before 29 weeks’ gestation, or HELLP syndrome diagnosed before 29 weeks. We have expanded the *Discussion* to state this inherent limitation of the study design more clearly.

**Discussion
Page 12, Lines 520-523**

Second, our findings regarding CBC-associated risk considered only uncomplicated pregnancies or those that developed complications after 29 weeks’ gestation, and therefore these results may not generalize beyond this target population to pregnancies with complications occurring prior to 29 weeks.

*(1.4) Can the authors comment on how they manage CBCs done at 26-29w if those with evolving HDP may have multiple tests drawn at that time--i.e which CBC was used.*

**Reply:** The reviewer is correct that repeated CBCs were performed in some individuals with evolving HDP. For this reason, we excluded individuals with elevated blood pressure prior to 29 weeks’ gestation, ensuring that the CBC included in the analysis preceded the first clinical signs or symptoms of the disease. In discovery, during the 26-29-week gestational window, only 2.3% of the considered pregnancies had more than one CBC; the majority of those had 2 CBCs, as shown in the figure below. Of repeat CBCs, 1.9% occurred in pregnancies without complications and 4.3% in pregnancies with complications. For our analysis, we used the first CBC recorded within the 26-29 window, and we have added this clarification to *Methods*.

**Methods**

**Page 5, Lines 177-178**

When multiple CBCs were present in any of the considered windows, we included the first chronological CBC for analysis.

(1.5) *It is known previously (as reference in the discussion) that hemoconcentration can be seen in patients with HDP.  In clinical practice, many signs are considered in the diagnosis of HDP.  These will include, as the authors are aware blood pressure readings, liver function tests, renal function tests, in addition to platelet counts etc.  Although clinicians are aware that hemoconcentration can also be seen, this variable might not be used often in clinical decision making.  Patients with ongoing anemia (some studies estimate that as many as 40% women are in pregnancy) might not "trigger" a clinician's suspicion of HDP if the Hb "normalizes".  The application of this finding into clinical setting might be limited.*

**Reply:** We agree entirely with the reviewer that, while clinicians may be aware of hemoconcentration, it is not current practice to consider it routinely, at least in part because routine markers or thresholds of hemoconcentration are not well-established. By identifying abnormal increases in red blood cell indices, our findings add to available evidence that incorporating these variables into obstetric decision-making may complement existing practices, particularly since CBCs are already routinely obtained at the time points we examined. Given our exclusion criteria, we are considering only pregnancies with complications in which the first symptoms of HDP appeared after the CBC, thereby making CBC risk factors additional potential first signs of hemoconcentration that could be incorporated seamlessly into clinical practice.

We agree that an increase in red blood cell markers following an anemia diagnosis and treatment may mask hemoconcentration or be misinterpreted as a risk factor. However, in the study cohort, ~83% of pregnancies identified as having a rare RBC dynamic were not anemic and maintained CBC values within established reference ranges at both 7–14 and 26–29 weeks’ gestation. These results indicate that these individuals do not meet clinical criteria for anemia. We have performed a sensitivity analysis evaluating positive predictive values in pregnancies with rare dynamics that either did or did not have clinical anemia and found that results were consistent in the two subgroups (**Table S18**).

**Results**

**Page 10, Lines 431-435**

Because iron supplementation initiated after a diagnosis of anemia at 7–14 weeks’ gestation could potentially influence CBC values, we also assessed the positive predictive value (PPV) of the identified thresholds separately in pregnancies with and without anemia. PPVs were consistent across these groups, indicating that the predictive performance of the thresholds was not driven by early anemia or its treatment (**Table S18**).

*(1.6) The authors mentioned that they excluded those with anemia in sensitivity analysis--how did the authors define anemia? Were the authors able to elicit the use of any Fe supplementation (oral or intravenous) in this discovery or validation cohort?*

**Reply:** We have used the ACOG definition of anemia [1]: hemoglobin <11g/dL during the first and third trimester and <10.5 g/dL during the second trimester. This definition is specified in the *Methods* section. We have now additionally extracted data for pregnancies with documented iron use from the discovery cohort and performed a sensitivity analysis of the reference-interval definition, evaluating them separately from those that were only clinically anemic. In our discovery cohort of 37,709 pregnancies without complications, N=1503 had iron supplementation recorded. This sensitivity analysis is shown below and also listed in *Supplementary Material* **Figure S4**.

**Methods**

**Page 5, Lines 189-194**

In sensitivity analyses, we determined gestational age-specific intervals in pregnancies without anemia[20,30] (HGB<11 g/dL at pre-pregnancy, 7-14 week’s gestation and prior to delivery, and HGB<10.5 g/dL at 26-29 weeks’ gestation), in pregnancies with IV or oral iron, and in the subset of term pregnancies without prior chronic conditions or pregnancy-related issues recorded in the EHR.

*(1.7) The manuscript was well-written.*

**Reply:** We thank the reviewer.

**Reviewer #2**

*(2.1) Thank you for the invitation to review this manuscript by Tozzo et al which aims to determine reference ranges for CBC in pregnancy and assesses how change during pregnancy is associated with perinatal outcomes. The team have utilized a large cohort and two distinct time periods to allow for discovery and validation which is a strength to the study.*

**Reply:** We thank the reviewer.

*(2.2) Overall, the results are difficult to follow, with reference ranges, many exposures, times points and outcomes. The authors should consider reducing exposures and sub-analyses. Although a correction for multiple testing has been applied type-1 error is still possible.*

**Reply:** We thank the reviewer for this helpful suggestion. While we do agree that Type I error remains possible even after Bonferroni correction, the replication of our key findings in an out-of-sample validation cohort substantially reduces the likelihood of false positives. We have expanded on this point in the *Discussion*.

Following the reviewer’s suggestion to focus on a smaller set of results, we have moved the results on SGA and transfusion to the supplementary material, reduced the scope of **Figure 3** and **Table 2**, and revised the *Results* section to improve clarity.

**Discussion**

**Page 12, Lines 513-514**

Fourth, our key findings were validated in an out-of-sample cohort, reducing the possibility of Type I error.

(*2.3) A major consideration is how adjusted models were selected. There are likely other factors that are confounders that should be considered - autoimmune disease, infections, maternal medication use, and smoking, which have not been considered or adjusted for. The potential for residual confounding is high and greatly undermines the interpretation of results.*

**Reply:** We thank the reviewer for pointing out the potential for residual confounding. As our analysis is designed to identify individuals at elevated risk rather than to infer causality, associations with blood markers retain clinical relevance even when influenced by confounding factors. To further evaluate the robustness of these associations, we conducted sensitivity analyses in the discovery cohort.. We ran a sensitivity analysis adding two binary variables indicating whether a pregnancy had any autoimmune conditions or an infection diagnosed during gestation. We also added a binary variable denoting whether the pregnant individual reported any smoking history, and we ran a sensitivity analysis restricting to pregnancies that made no use of medications throughout their pregnancies or took medications only from list that we deemed unlikely to affect CBCs (list available in *Supplementary Methods*). Results show that the prevalence of complications is similar across these subgroups (**Table S1**), and the odds ratios are consistent with those from the main analysis (**Figures S7** and **S8**). We have added this analysis in *Methods* and *Results*.

**Methods**

**Page 7, Lines 269-280**

To evaluate the robustness of the reported associations, we conducted three sensitivity analyses within the discovery cohort. First, we included pregnancies that had been excluded in the primary analysis due to stillbirth or underlying blood disorders. Stillbirths were added to the composite outcome, and the presence of a blood disorder was added to our GEE model as a covariate. Second, we additionally adjusted for covariates capturing potential immune- and inflammation-related confounding, including pre-existing or incident autoimmune conditions and infections during pregnancy (ascertained via Phecodes; see **Text S11**), as well as past or current smoking status. Third, we restricted the analysis to pregnancies with no medication exposure or with exposure limited to medications not known to affect hematological dynamics (see **Text S12** for the full medication list). For all three sensitivity analyses, associations between outcomes and either out-of-range CBC values or rare dynamics were tested and compared to associations from the primary analysis.

**Results**

**Page 9, Lines 373-377**

Sensitivity analysis in the discovery cohort including pregnancies resulting in stillbirth or reporting blood disorders (**Table S10**), adding autoimmune conditions, infections, and smoking status (**Table S11**), or restricting to those pregnancies that did not report medications possibly affecting CBC dynamics (**Table S12**), showed consistent results with the primary analysis (**Figure S7)**.

**Page 10, Lines 425-430**

We evaluated the robustness of the thresholds identified in the discovery cohort through three sensitivity analyses. These included: (1) expanding the cohort to include pregnancies complicated by stillbirth or blood disorders (**Table S15**); (2) additionally adjusting for autoimmune conditions, infections, and smoking status (**Table S16**); and (3) restricting the analysis to pregnancies not exposed to medications that could plausibly affect CBC dynamics (**Table S17**). Across all analyses, results were consistent with the primary findings (**Figure S7**).

*(2.4) Additionally, how were blood disorders handled in the validation cohort? These were excluded in the discovery cohort but not validation.*

**Reply:** We thank the reviewer for the question. Both in discovery and validation, we excluded blood disorders based on diagnoses in the medical record problem list, which were derived from text-based fields and ICD codes. We have clarified this approach in the *Methods*, and a full list is provided in the *Supplementary Methods***.**

**Methods**

**Page 4, Lines 151-158**

In both discovery and validation cohorts, we included singleton pregnancies ending in live birth and excluded those with known blood-related disorders (including pre-existing diagnosis of immune thrombocytopenia purpura, hemolytic anemia, or a genetic blood disorder based on available diagnoses in the problem list or ICD9/10 codes, see **Text S1** for details), with known chronic hypertension or elevated blood pressure prior to 20 weeks’ gestation (see **Text S2** for details), who delivered prior to 29 weeks’ gestation (required for determining exposure), or who were missing data on birth weight or neonatal sex (required for determining a primary outcome).

*(2.5) Why do inclusion/exclusion criteria differ between the validation and discovery cohorts? This needs further justification.*

**Reply:** We appreciate the reviewer’s feedback regarding the alignment of our cohorts. We have revised the inclusion/exclusion criteria for the validation cohort to align with those of the discovery cohort and maximize comparability between the two groups.

The sole remaining difference now is whether the pregnancies receiving transfusions during pregnancy were excluded or not. This exclusion was essential in the discovery phase to establish baseline reference ranges and dynamics in healthy pregnancies (**Figure 3**). We found that transfusions were not significantly associated with CBC parameters, which were the primary focus of our validation analysis (**Tables S7, S9, S13 and S14**). To maximize the sample size and power of the validation cohort, we retained these individuals. We have updated **Figure 2** (Figure 1 in the previous version), *Supplementary Material*, and all downstream results to reflect these redefined criteria.

*(2.6)   Projected models may introduce significant bias and do not form the main analysis, the author should consider removing this.*

**Reply:** We thank the reviewer for the suggestion. We have now removed the projected models from the paper for ease of reading and updated **Table S4** and **S6** and text in *Results* accordingly.

*(2.7) It is unclear how 'rare' changes were defined and must be updated. Is it possible that these rare values are outliers/errors in ascertainment/analysis?*

**Reply:** We thank the reviewer for this comment. We have updated the *Methods* to clarify our definition of rare changes and introduced a visual depiction (now **Figure 1**) to facilitate reading.

Measurement errors and artifacts are always possible, but they are unlikely to have influenced our results regarding rare changes, because for most of the identified rare changes, the starting and ending CBC results fell within established reference intervals. For example, for the rare changes with the strongest associations with outcomes (HCT, HGB, RBC, PLT) CBC results fell within established reference intervals for 78-95% pregnancies at 7-14 weeks and 26-29 weeks’ gestation. It is also important to note that the institutional reference intervals used at Massachusetts General Hospital remained unchanged throughout the study period, and all hematological analyzers were in rigorous internal quality control and external proficiency testing programs throughout the study period.

*(2.8) How was missing data handled? It's unclear from the tables what proportion of missing data was in the dataset. What does extrapolated based on population model mean for missing BMI? This is not how missing data is commonly handled and is confusing given the authors have also stated that when BMI was left as missing if not recorded (legend for Table 1).* 

**Reply:** We thank the reviewer for identifying these areas for clarification. We have revised the text to reflect our approach. We do not have any missingness in CBC parameters, which are our primary exposures. Missingness across all covariates (Age, Parity, Race/Ethnicity, BMI, etc) was low, accounting for only 3.7% in discovery and 10.7% in validation. In primary analysis, we required complete cases.

We apologize for the confusion regarding BMI. To maintain a consistent timepoint of 12 week’s gestation across all pregnancies, we utilized a validated model[3] to estimate BMI for pregnancies whose measurements were taken outside the immediate 12-week window. This approach is a form of multiple imputation that depends on available data rather than imputing with the population mean. We have added more detail in *Supplementary Material* and updated the validation cohort to use the same variable for uniformity.

**Methods**

**Page 6, Lines 235-244**

Associations quantified via odds ratios (ORs) were then computed with generalized estimating equations (GEE) for logistic regression in order to account for the lack of independence between pregnancies in the same individual.[32–34]. Clustering was enforced at the level of the individual and an exchangeable correlation structure was used. Models were adjusted for 1^st^ trimester BMI, age, insurance status, race/ethnicity, and parity. Missingness was low across covariates (3.7% in discovery and 10.7% in validation) and complete cases were used for association testing

*(2.9) The authors have not included the number in each group/analysis, this should be added throughout. Given <10% had all time points it is likely the numbers are low for several analyses.*

**Reply:** We thank the reviewer for pointing out this oversight. We want to highlight that only the analysis focused on longitudinal changes had a smaller number of included pregnancies (N=2,791), and results for this analysis were replicated in the sensitivity analysis that included all available pregnancies. For association analyses, we analyzed N=34,159 pregnancies in the discovery and N=48,490 in the validation. We have now included the sample sizes for each analysis in the Results section.

**Results**

**Page 7, Lines 306-309**

To derive new reference intervals, we analyzed distributions of CBC indices in pregnancies without complications in the discovery cohort (N=37,709) and compared trimester-specific CBC index reference intervals to those in commonly-cited literature and to those in regular use for non-pregnant adult females (**Figure 2**).

**Page 8, Lines 320-322**

In our analysis of the subset of the uncomplicated term pregnancies that had all CBCs available at the 4 examined timepoints (including prior to pregnancy and 3 pregnancy timepoints, N=2,791)

**Page 8, Lines 339-341**

In discovery, we investigated the use of the 26-29 weeks’ gestation reference intervals for the identification of pregnancies at elevated risk of subsequent adverse outcomes. Of those N=34,159 pregnancies with and without complications having a CBC at 26-29 weeks’ gestation, pregnancies with HGB, HCT, or RBC above the reference interval at 26-29 weeks’ gestation were significantly more likely to have the composite outcome (**Figure 4, Table S7**)

**Page 9, Lines 362-363**

Results in the validation cohort (N=48,490) were consistent with those in the discovery cohort (**Figure 4, Table S9**).

**Page 9, Lines 382-384**

Given the large variation in CBC indices during pregnancy, we tested whether rare changes in CBC indices were associated with the risk of subsequent complications in participants who had a CBC at 7-14 and 26-29 weeks’ gestation (N=29,416 discovery, N=48,490 validation).

*(2.10) How were changes to diagnostic criteria for PE handled over the study period and different time points?*

**Reply:** We appreciate the reviewer’s concern regarding potential changes in clinical guidelines for preeclampsia (PE) diagnosis over time. Because our primary objective was to identify a consistent biological signal, we applied a unified PE definition across all pregnancies in both the discovery and validation cohorts (see Methods and Supplementary Methods). This definition was validated in the discovery cohort (Maternal Health Cohort) through blinded chart review by maternal–fetal medicine specialists, achieving an accuracy of 90%. We acknowledge that temporal changes in clinical practice and management of PE may introduce residual confounding. Accordingly, we have explicitly addressed the potential impact of temporal effects as a limitation in the Discussion.

**Discussion**

**Page 12, Lines 531-534**

Fourth, discovery and validation cohorts spanned different time periods with evolving practices and diagnostic coding, leading to differences in prevalences and possible misalignment. However, the consistent replication of associations across cohorts suggests that the identified biological signal is robust.

*(2.11) Reference intervals are not described in the results*

**Reply:** We thank the reviewers for this comment. We have added a sentence in the Results describing the consistency of the reference intervals for HCT, HGB, WBC, PLT, and RDW, and made a more explicit reference to **Table S3**, where all the intervals are listed. We elected not to present all individual reference interval values in the main text to maintain readability consistent with the feedback to reduce the number on main text results, but we can easily move them if the editors and reviewers prefer.

**Results**

**Page 7, Lines 306-311**

To derive new reference intervals, we analyzed distributions of CBC indices in pregnancies without complications in the discovery cohort (N=37,709) and compared trimester-specific CBC index reference intervals to those in commonly-cited literature and to those in regular use for non-pregnant adult females (**Figure 2**). All numerical values are provided in **Table S3.** The reference intervals for HCT, HGB, WBC, PLT, and RDW were consistent with previously reported ranges.

*(2.12) Abstract is lacking results on defining reference ranges*

**Reply:** We have added a sentence to highlight the reference ranges.

**Abstract**

**Page 2, Lines 70-73**

Derived reference intervals differed from those in the literature for mean red cell volume, mean red cell hemoglobin, red cell count, and mean red cell hemoglobin concentration; reference intervals for other indices were similar to those previously published.

*(2.13) The introduction could be expanded to describe the longitudinal changes and associations found outside of pregnancy*

**Reply:** We thank the reviewer for this thoughtful suggestion. The Introduction mentions this important prior work on CBC-related associations and longitudinal changes outside of pregnancy. We would be happy to elaborate further if the editors and reviewers recommend. We look forward to follow-up studies specifically designed to investigate these interesting connections regardless.

*(2.14) Is the third time point simply >36 weeks? (described in methods as up to 7 days before 37 weeks).*

**Reply:** The third time point in pregnancy was pre-delivery, and it was defined based on the delivery date for each pregnancy. We considered the CBC closest to delivery within a 0 to 7 days period. We have updated the *Methods* to reflect this more clearly.

**Methods**

**Page 5, Lines 171-174**

Based on the timing of routine CBC screening (**Figure S2**), we considered three pregnancy timepoints: 7-14 weeks’ gestation (7 weeks 0 days to 14 weeks 0 days), 26-29 weeks’ gestation (26 weeks 0 days to 29 weeks 0 days), and pre-delivery, which was pregnancy-specific and defined as the CBC closest to delivery within a 0 to 7 days window.

*(2.15) Maternal clustering is described in the supplement but details of this should be included in methods.*

**Reply:** We thank the reviewer for this critique, and we have added a line to address this concern in the Methods section.

**Methods**

**Page 6, Lines 236-239**

Associations quantified via odds ratios (ORs) were then computed with generalized estimating equations (GEE) for logistic regression in order to account for the lack of independence between pregnancies in the same individual[32–34]. Clustering was enforced at the level of the individual and an exchangeable correlation structure was used.

*(2.16) Results of validation should be discussed and included in the main text and described how they are similar or differ to discovery*

**Reply:** We thank the reviewer for the suggestion. We have now added two paragraphs describing the validation including replication results for out of range odds ratios and rare dynamics.

**Results**

**Page 9, Lines 362-**372

Results in the validation cohort (N=48,490) were consistent with those in the discovery cohort (**Figure 4, Table S9**). In particular, pregnancies with HGB, HCT, or RBC above the reference interval at 26-29 weeks’ gestation were significantly more likely to have the composite outcome: OR 1.4 (95% CI [1.2, 1.5] p<0.0001) for HCT, 1.6 (95% CI [1.4, 1.8] p<0.0001) for HGB, and 1.6 (95% CI [1.4, 1.7] p<0.0001) for RBC. Elevated HGB , HCT, and RBC at 26-29 weeks’ gestation were associated with HDP with odds ratios for HCT of 1.5 (95% CI [1.3, 1.6], p<0.0001); for HGB 1.7 (95% CI [1.4, 2.0], p<0.0001); and for RBC 1.5 (95% CI [1.3, 1.7] p<0.0001). HGB and RBC above range were also consistently significantly associated with preterm birth (HGB: 1.4 (95% CI [1.1, 1.9], p=0.0076); RBC:1.6 (95% CI [1.3, 1.9] p<0.0001). RBC below range was significantly associated with reduction in HDP (0.7 95% CI [0.6, 0.8], p<0.0001).

**Page 9, Lines 399-408**

Consistent results were also found in the validation cohort using the thresholds defined in the discovery cohort (**Table 2, S14**). For the composite outcome, an increase of >1.8% in HCT was significantly associated with an OR of 1.5 (95% CI [1.3, 1.7] p<0.0001) , as was an increase of >0.67 g/dL in HGB, with an OR of 1.5 (95% CI [1.3, 1.8] p<0.0001), an increase >0.07 10^6^/mm^3^ in RBC had an OR of 1.7 (95% CI [1.5, 2.0] p<0.0001), a decrease of less than 0.05 10^3^/µl or an increase in PLT had an OR of 1.09 (95% CI [1.04, 1.15], p=0.0008), and a decrease in MCV greater than 0.75 fL had an OR of 1.3 (95% CI [1.2, 1.4], p<0.0001). For the majority of pregnancies with complications identified by these rare changes, the relevant CBC index value was within the 26-29 weeks’ gestation reference interval (71-93%) and was therefore unremarkable on its own (**Table 2**).

*(2.17) Figure 3 is difficult to understand. The discovery and validation OR and 95% CI should be presented individually as per the methods.*

**Reply:** We thank the reviewer for the suggestion. In response, we have updated Figure 3, now Figure 4, as requested, including by moving results for SGA and Transfusion to Table S7. To focus on the most important results, we also removed the two-sided out-of-range exposure since we deemed it less clinically meaningful than specifically above or below; this analysis remains in the supplemental tables. We have added the OR and 95% CI of the validation cohort, and have marked those associations that were nominally significant.

**Reviewer #3**

*(3.1) This is a well-written manuscript with robust statistics, I have no recommendations for revisions*

**Reply:** We are very pleased that the reviewer feels our analysis is robust and that our report is well-written.

**Reviewer #4**

(*4.1) This is a valuable contribution on the haematological changes associated with pregnancy.
The normal range data largely confirms the comparative reference for Hb and Hct, with some greater discrepancies for some of the RBC indices, presumably due to the different methodologies employed.*

**Reply:** We appreciate that the reviewer feels our manuscript is a valuable contribution.

*(4.2) The relatively lower value of diminished/diminishing haemoglobin/haematocrit values in pregnancy compared to increasing values is perhaps surprising  and deserves a little bit more of discussion.*

**Reply:** We thank the reviewer for highlighting this point. Several prior studies have reported associations between lower hemoglobin or hematocrit levels and obstetric complications; however, results are mixed in regard to the timing of when these associations are strongest [7–10]. Some work has demonstrated lower hemoglobin values are more strongly associated with adverse outcomes in the first trimester [11,12]. Given our narrow exposure window, the timing difference may explain why we did not observe these associations in our analyses. We have updated the Discussion to emphasize that the timing of assessment plays an important role in these relationships and may explain discrepancies between our findings and prior work.

**Discussion**

**Page 11, Lines 478-485**

Current guidelines on CBC interpretation during pregnancy focus primarily on low values, which in our analysis were not significantly associated with adverse outcomes. Previous studies suggest that the timing of assessment may influence observed associations[17,50]. Our results, derived from a narrower gestational window than most previous studies, indicate that it might be beneficial to consider high CBC values at this specific stage of pregnancy as early indications of subsequent complications. Because CBCs are already routinely collected, incorporating evaluation of high values may offer a cost-effective approach to help detect high-risk pregnancies.

*(4.3) The use of three different haematology analyzers and two if not three different technologies is a substantial limitation of this work and may explain the differences reported here for some of the red cell parameters compared with other data sets. It is important to convince the readers that this methodological heterogeneity is not causing some of your findings. Can you provide sub-analysis which  can address these concerns? For example is your 2016-2022 Sysmex dataset different for some of the RBC parameters  with the Advia one obtained prior to 2012? Same for the Sysmex He-5000 vs Sysmex HN-9000, since some of the RBC technology differs in these systems.*

**Reply:** We appreciate the reviewer’s attention to this technical detail and the opportunity to discuss this important issue more in depth. We agree that cross-instrument variability is a potential confounder in longitudinal studies. To address this possibility, prior to analyses, we conducted a systematic evaluation of parameter stability across the three analyzers. All parameters demonstrated consistency across instruments except Mean Platelet Volume (MPV), see figure below, which exhibited significant bias across analyzers, possibly due to the lack of inclusion of MPV in standard external proficiency testing programs. Consequently, MPV was excluded from all subsequent analyses. It is important to note that the institutional reference intervals used at Massachusetts General Hospital remained unchanged throughout the study period even when instruments methodology or models were changed. We added the figure below as a supplementary figure and added a reference to this analysis in **Methods**.

**Methods**

**Page 5, Lines 166-170**

We considered the following CBC indices: hematocrit (HCT), hemoglobin (HGB), white blood cell count (WBC), red cell count (RBC), platelet count (PLT), mean red cell volume (MCV), mean red cell hemoglobin content (MCH), red cell distribution width (RDW), and mean red cell hemoglobin concentration (MCHC). See **Text S2** for detail on measuring instruments. We did not consider mean platelet volume due to shifts across hematological analyzers (**Figure S1**).

**Figure S1**

*(4.4) Please discuss how your CBC methodologies compare with the comparative reference (Maternal Health Cohort ). Please discuss if there are notable differences between the two data set in methodologies.*

**Reply:** We thank the reviewer for this comment. We have made every effort to harmonize methodologies between the discovery (Maternal Health Cohort) and validation cohorts to ensure a direct comparison. We have now aligned the inclusion/exclusion criteria across both datasets and applied the same validated BMI-imputation algorithm to both datasets. The laboratory methodologies (CBC extracted from EHR) were identical, and as shown in the previous response 4.3, instrument transitions in time did not introduce significant bias in the reported parameter studies.

**Discussion**

**Page 12, Lines 531-534**

Fourth, discovery and validation cohorts spanned different time periods with evolving practices and diagnostic coding, leading to differences in prevalences and possible misalignment. However, the consistent replication of associations across cohorts suggests that the identified biological signal is robust.

(*4.5) I could not see any reference to ethnicity and age for the studies cohort*.

**Reply:** We thank the reviewer for this comment. We have now added two sentences in results related to age and ethnicity. This information is also provided in Table 1 for both the discovery and validation cohorts.

**Results**

**Page 7, Lines 298-303**

We analyzed the demographic characteristics of pregnancies that were excluded from the analysis (**Table S2**), which show a higher number of pregnancies with public insurance, a higher number of individuals who self-identified as Black or Hispanic, and a higher BMI. Their exclusion could be due in part to socio-economic factors that led to reduced access to care and prevented us from collecting necessary CBC data for the analysis.

**Page 8, Lines 356-360**

Among the covariates, maternal age, BMI, and public insurance status were at least nominally significantly associated with an increased risk of the composite complication, with modest effect sizes. In contrast, parity showed a strong and consistently protective association. No significant associations were observed for race and ethnicity. Effects were consistent across CBC parameters and ranges (**Table S8**).

*(4.6) It would be valuable to have more clinical data on the pregnancies with complications, especially blood pressure values and how they may relate to changes in hematological parameters. Same for renal function and at least proteinuria. The interpretation of the significance of the noted hematological changes is a challenge in the absence of more granular clinical data outside of the outcome.*

**Reply:** We agree with the reviewer that more granular clinical data would be valuable for interpreting the observed hematological changes. However, by design, we excluded all individuals with evidence of complications at the time of CBC assessment. Consequently, abnormalities in blood pressure, renal function, and liver function would be expected to emerge or be evaluated after the exposure window considered in this analysis. As such, examining these measures falls outside the scope of the current study, which focuses on evaluating CBC parameters as early risk factors for subsequent obstetric complications.

*(4.7) I would like to have a more in depth discussion of PLT changes, especially if they appear int he absence or presence of other CBC changes. What are the characteristics of patients with decreasing PLT count and their susceptibility t o complications of pregnancy?*

**Reply:** We thank the reviewer for this comment. In our analysis, platelets trajectories showed a significant association with composite outcomes. Specifically, an increase in platelet count between 7-14 weeks and 26-29 weeks’ gestation was associated with an odds ratio of 1.2 [1.12, 1.29], p < 0.0001. Out-of-range platelet values showed nominally significant associations in discovery and Bonferroni significant in validation with an odds ratio of 1.25, 95% CI [0.06, 1.48], p=0.009 for the composite outcome and 1.38 [1.09, 1.75], p=0.007 for hypertensive disorders of pregnancy (Table S7 and S11). These findings are consistent with a prior large study [Reese at al, 2018] which found that pregnancies with HDP exhibit elevated platelet counts through approximately 32 weeks of gestation. This pattern is reversed as pregnancies approach delivery, which is consistent with the observed low platelet characteristics of HELLP syndrome seen in previous findings. Our results replicate these findings. We have added a sentence to the *Discussion* to emphasize this point.

While a more detailed evaluation of platelet changes in conjunction with other hematological parameters could further elucidate patient sub-phenotypes and susceptibility to complications, such multivariate analysis is beyond the scope of the current study. We have added text to the Discussion noting this as an important direction for future work.

Reese, J. A. *et al.* Platelet Counts during Pregnancy. *N Engl J Med* **379**, 32–43 (2018).

**Discussion**

**Page 11, Lines 476-478**

We also found that, although not significant after multiple testing, elevated PLT count was nominally significantly associated with the composite outcomes and HDP, in line with previous findings on platelets dynamics during pregnancy[17].

**Page 12, Lines 534-536**

Fifth, we only evaluated univariate associations between CBC and obstetric complications, and it is possible that a multivariate approach could lead to stronger findings.

**Editorial requests**

(R.1) We ask every co-author listed on the manuscript to fill in a contributing author statement, making sure to declare all competing interests. If any of the co-authors have not filled in the statement, we will remind them to do so when the paper is revised. If all statements are not completed in a timely fashion this could hold up the re-review process. If new competing interests are declared later in the revision process, this may also hold up the submission. Should there be a problem getting one of your co-authors to fill in a statement we will be in contact. Please do not add or remove authors without first discussing this with the handling editor. You can see our competing interests policy here: <http://secure-web.cisco.com/1riKpCqprhf-Xfvw2zhzsnsxMrIs4txEvVC5wDWaQw83R2alojQKiU_iXo1Q5-8K6uzlvI0aNO8v8ePl0QIHFLcVv7SKbUome8wABAXZ1db9eR4XjZ1JkypqrLEABCq9XVpX5KTXNrl8e60TxUF5YbimE_vC9QoS9veBETG01_Odn8bXsMjwqmjSuvZ33let_3U6V4YpCMwdms2mS3yCC3JNH8SRZjMWPdFjuzKAWYCc3YbvXgaB2sW_MFqaSVQQGjlG0SQyd42mcjqKaEOcGC5O4ySFtwRsWElD8G62cluuDMbDZ4gEe9LCuAAGLz5_d/http%3A%2F%2Fjournals.plos.org%2Fplosmedicine%2Fs%2Fcompeting-interests>

(R.2) Please upload any figures associated with your paper as individual TIF or EPS files with 300dpi resolution at resubmission; please read our figure guidelines for more information on our requirements: <http://secure-web.cisco.com/1D6mstyzDuiwYbc6JZacuo8HH-A9zREgAaeCfRegEa_enWNEOmjw2kwf8S_eNH3VAzONyE5bxZpIXfh-5_8bj-hCtPWY38UoexZZftexk5Dp6yymon5Syox6GEJVT6-wf9IDOY_0zKG9C3CKkg-qr3gunmcHlX1v0UOls9XjVXeqYL_ji7Xpfd78vPAL993w0-sfBmMBoui0I09Ppo6M5OYrs5pvgZs7QbwkC4p9sj2r02AH3RPZsBRtCve4NnWUJ4KLXrxIsk0LlGq3-eJaQqYV5B2MUrsz1RjcutqGtf_FQhDjx1R0rZEOHn7xEICIA/http%3A%2F%2Fjournals.plos.org%2Fplosmedicine%2Fs%2Ffigures> While revising your submission, we strongly recommend that you use PLOS's NAAS tool (<https://secure-web.cisco.com/1Qjpw4EQSwn2Yi5EBCwC76tYWOWw_4bsCfmm4cZ2cMD7IGi4JgFSa_59LoODVpWxMAmmgDuYOPE_Fs9_HenG41yxpjq5K8ildsbxzZWb_kECFm26iY1vRyELNJxv50cKRB0IuUyJiT3V_vYcai8RSBDKV1SA88NOC4Z1cONYuidiKfjHdUsTYhljQ5PuuBf7Ulqb8KIq1Q54pHrdiEKMtC7AbXF1wJBuYaK8picuszCsKr9omD7h-ubaju5zXtGMCkenhvmbifzh3yj3yc-DLvzH13wfkZzo34FzuVxtXSpUrZCT-RvnufqczGiS1fpLb/https%3A%2F%2Fngplosjournals.pagemajik.ai%2Fartanalysis>) to test your figure files. NAAS can convert your figure files to the TIFF file type and meet basic requirements (such as print size, resolution), or provide you with a report on issues that do not meet our requirements and that NAAS cannot fix.

After uploading your figures to PLOS's NAAS tool - <https://secure-web.cisco.com/1Qjpw4EQSwn2Yi5EBCwC76tYWOWw_4bsCfmm4cZ2cMD7IGi4JgFSa_59LoODVpWxMAmmgDuYOPE_Fs9_HenG41yxpjq5K8ildsbxzZWb_kECFm26iY1vRyELNJxv50cKRB0IuUyJiT3V_vYcai8RSBDKV1SA88NOC4Z1cONYuidiKfjHdUsTYhljQ5PuuBf7Ulqb8KIq1Q54pHrdiEKMtC7AbXF1wJBuYaK8picuszCsKr9omD7h-ubaju5zXtGMCkenhvmbifzh3yj3yc-DLvzH13wfkZzo34FzuVxtXSpUrZCT-RvnufqczGiS1fpLb/https%3A%2F%2Fngplosjournals.pagemajik.ai%2Fartanalysis>, NAAS will process the files provided and display the results in the "Uploaded Files" section of the page as the processing is complete.
If the uploaded figures meet our requirements (or NAAS is able to fix the files to meet our requirements), the figure will be marked as "fixed" above. If NAAS is unable to fix the files, a red "failed" label will appear above.
When NAAS has confirmed that the figure files meet our requirements, please download the file via the download option, and include these NAAS processed figure files when submitting your revised manuscript.

(R.3) Please ensure that the paper adheres to the PLOS Data Availability Policy (see <http://secure-web.cisco.com/1b7hAJI25RhyDV2nbtdOnOADb3BNXQPtSlfpTxHQWTtzUwTLHNlNTCEcIE37OGvrOs-nbN_SZE33PINbV_WqmaFBgn1fUwDPFDfwvyiyQymmZctDaJ0XwJ31_vR-DXvWEZJQDyJX7BjFbxKzORCyRBtp31NPyd2kQG8X1zTCp2hrZcCTAFMEBTt02OJWNsEsZqoc4BGonn3mMSTgTdYvH4LRB1ZJ0_kEt0qj_G1sz3tDunRtITB7rfpzBhy-RUWyVu33POI1lzQBR48s1KVS_XVvGxTskKYHhO2QnTO9SI5XVb8Ey2-z8oKkwnUPH1zg-/http%3A%2F%2Fjournals.plos.org%2Fplosmedicine%2Fs%2Fdata-availability)>, which requires that all data underlying the study's findings be provided in a repository or as Supporting Information. For data residing with a third party, authors are required to provide instructions with contact information (web or email address) for obtaining the data. Please note that a study author cannot be the contact person for the data. PLOS journals do not allow statements supported by "data not shown" or "unpublished results." For such statements, authors must provide supporting data or cite public sources that include it.

(R.4) Thank you for including a data availability statement. As these data cannot be made public, please include details on how researchers could gain access to these data.

**Reply**: The statement is now updated in the text.

(R.5) Please include the IRB approval number in your ethics statement.

**Reply**: We have now added an ethics statement with the IRB numbers.

(R.6) Financial disclosure: Please include URLs to the funders.

**Reply**: We have one so in the Funding and Assistance paragraph.

FORMATTING – GENERAL

*(R.7) Abstract: Please structure your abstract using the PLOS Medicine headings (Background, Methods and Findings, Conclusions). Please combine the Methods and Findings sections into one section.*

**Reply:** Thank you for pointing out the discrepancy. We have now updated the abstract to match PLOS Medicine guidelines.

(R.8)  At this stage, we ask that you include a short, non-technical Author Summary of your research to make findings accessible to a wide audience that includes both scientists and non-scientists. The Author Summary should immediately follow the Abstract in your revised manuscript. This text is subject to editorial change and should be distinct from the scientific abstract. Ideally each sub-heading should contain 2-3 single sentence, concise bullet points containing the most salient points from your study. In the final bullet point of 'What Do These Findings Mean?', please include the main limitations of the study in non-technical language. Please see our author guidelines for more information: <https://secure-web.cisco.com/1cQlOAEx3oChkS5WJyEoWHoIWQNbdRmBsR2hyiroo_VTunuqjxUXVwnHsEWjzKYraLHGadyP7atuRv97NId6oNEdpBmPEKvckQlkRHau08j-9293jySuX1twfa4DGrusoCZ3oidE1V8sJNdVMe_ipspCHjoRnW9lKb6WdTTFxuEV9r3S5BcH-Grn92lMaGhpM4fSWAxAvzW9TXraI1PJesODDQWrlSN-gToTmBvWEW4WQMhvva310rdi2GVlnkYOOvZrDceVmXLcJgFPOiBg9S3hYMGlir3zZqZGvcOT3gQx9VQAM8h7qAi0ytlDgEIWk/https%3A%2F%2Fjournals.plos.org%2Fplosmedicine%2Fs%2Frevising-your-manuscript%23loc-author-summary>

**Reply**: The Author summary is now added after the abstract.

(R.9) Please express the main results with 95% CIs as well as p values. When reporting p values please report as p<0.001 and where higher as the exact p value p=0.002, for example. Throughout, suggest reporting statistical information as follows to improve clarity for the reader "22% (95% CI [13%,28%]; p</=)". Please be sure to define all numerical values at first use.

**Reply**: We have now changed how we report these values. We have chosen to report p<0.0001 as we use a Bonferroni threshold of 0.0003 for some of our findings, and a p<0.001 would be above such a threshold.

(R.10) Please include page numbers and line numbers in the manuscript file. Use continuous line numbers (do not restart the numbering on each page).

**Reply**: We have added numbering.

(R.11) Please cite the reference numbers in square brackets. Citations should precede punctuation.

**Reply**: We have now changed the style.

FIGURES AND TABLES

(R. 12) Please provide titles and legends for all figures and tables (including those in Supporting Information files).

**Reply**: The legends are provided at the end of the main document.

(R.13) Please define all abbreviations used in each figure/table (including those in Supporting Information files).

**Reply**: We have now added abbreviations in captions or footnotes.

(R.14) Please consider avoiding the use of red and green in order to make your figure more accessible to those with color blindness.

**Reply**: We thank the editor for this suggestion. We are happy to revise the figures to avoid the color combinations. If there are any specific figures that the editor would like us to modify, we would appreciate that guidance.

(R.15) Please cite your Supporting Information as outlined here: <https://secure-web.cisco.com/14-3U6cs9w31B8bBJ1jwnsyeKqMoYzJE9T1Po-kGPc2I5J8XltoDyIe3IGULe-l9O41l3Oh4_uXS20WgR7DZJDKhIuZbq-GwBa2J6oPJaFt7UiZ639WBp8UTI-sCZ_kxx0jGOT4e1zlOEHg3sPikp9HOvQPnh-kFUZOlKXvw44TZd5qJQselap0g7UWa0o-r2flrkS6zdQMXHhZ5uN2xxv4HaxBLKZIjDH9KhABfBn33SLjvpXUQgxDxxUzNamCtbEiuyLHBVD7ESiHUyJDWFpypJ3pIrZ_sWrInqVXjx4_aO6OONNsoAWtebJhb46AB2/https%3A%2F%2Fjournals.plos.org%2Fplosmedicine%2Fs%2Fsupporting-information>

**Reply**: We have now updated references to supporting information.

REFERENCES
*       PLOS uses the numbered citation (citation-sequence) method and first six authors, et al.

**Reply**: We have updated the references.

*       Please ensure that journal name abbreviations match those found in the National Center for Biotechnology Information (NCBI) databases (<http://secure-web.cisco.com/1O7r-ueem6bRmbzUjGb4MMNuhQx-ZdSjTbATwD1FkrVDt4IzozCr-4wpkZ0P137xelqF1Le2a80LzDj6PP-aBpG6fC9acXf1IQhpNdgYVp5vL8ghuV5gol6wgkkcaxN3w9I-pW5WCM79_VOqproLUaJv3hSrzCxXH6X48HXEVMgfB9ZsjzKzTKpdsZbRF7Pdl1lo3sZpzSRxDaPZsx8wtyugYI4DJoVkoivALUZUIc2OHJoAiFPoEPy6fLLSQTqNKkj2bd9IymCvo_kU2ip6S0jaEmf9wjABcIIihHRDIjM896IoKfWMybiuhCSvOhT8d/http%3A%2F%2Fwww.ncbi.nlm.nih.gov%2Fnlmcatalog%2Fjournals>), and are appropriately formatted and capitalised.

**Reply**: We have performed this check.

*       Where website addresses are cited, please include the complete URL and specify the date of access (e.g. [accessed: 12/06/2023]).

**Reply**: We have performed this check.

*       Please also see <https://secure-web.cisco.com/1GzJpT0FV6dCr92owuB8E4b80_XMlM-UNpYlRkc6wzbe6N7Hx5k_56hzI1zH0wwTtns5Y1oFbb9qb4OYo_wUnlHrGhXHRp8nfB5eHsqay48U3O8y1woaRGnAOmfbS8Hk0fa9oRAuzoDLwaLqFXJgbINBcQJyml1CrIlYpSoq7VXOZd3s0WGLOoKZA1QIFPQ4NsQdLHn0jhyGRRG1r6kX9c02E2UqDNG7ZqFnB8YZT_2tRHrAwsXxT35CS2JoCNNVCHZOZtv7oMJcWD2uVFhilS94Sl3pmWIZaWWZKmS1VCvrTRHH4SBmJuqNCH1z18C9i/https%3A%2F%2Fjournals.plos.org%2Fplosmedicine%2Fs%2Fsubmission-guidelines%23loc-references> for further details on reference formatting.

OBSERVATIONAL STUDIES

*       Abstract: Please include the study design, population and setting, number of participants, years during which the study took place (enrollment and follow up), length of follow up, and main outcome measures.

**Reply**: We have updated the abstract to match PLoS Medicine guidelines, and it now includes all this information.

*       [FOR POPULATION HEALTH/REGISTRY STUDIES] Please ensure that the study is reported according to the RECORD guideline (available from <https://secure-web.cisco.com/1dJ4_V3KdDUSoTfuKHKT8GbC0_ZDceY7CxzoKwhFVNYljMgE-tz9NDAE2TQR626pfVT0Ee14xkTLatkzKFXoqikjqpglf52uL52wkjTwrLq3NyO-CA73JA2zh8P4PEm05tPTIh-AM5C64e6ZlEFFm0UyF8vYXdFrdGKGbh2KhLy0TkhZHANDUWQp27Q_r8VwAWOHhJjdXjXGBHtHz9QngMN02eCSPgshS1MqhKuRLKhc3JFOTHbCx7bPXLMRE2j4IHBR82O2U_PBJCJpF4HGl74SDKo0j2rHHD--FIY5T2q55sFTap0y1lJO1soLKS8sY/https%3A%2F%2Fwww.record-statement.org)> and include the completed checklist as Supporting Information. Please add the following statement, or similar, to the Methods: "This study is reported as per the Reporting of Studies Conducted using Observational Routinely-Collected Data (RECORD) guideline (S1 Checklist)." When completing the checklist, please use section and paragraph numbers, rather than page numbers.

**Reply:** We have added the checklist as Supplementary Information

*       For all observational studies, in the manuscript text, please indicate: (1) the specific hypotheses you intended to test, (2) the analytical methods by which you planned to test them, (3) the analyses you actually performed, and (4) when reported analyses differ from those that were planned, transparent explanations for differences that affect the reliability of the study's results. If a reported analysis was performed based on an interesting but unanticipated pattern in the data, please be clear that the analysis was data driven.

**Reply**: We have updated the methods based on the reviewers’ feedback and these elements are now present. We are happy to provide further updates if there are any specifics that you would like us to change.

*       Please state in the Methods section whether the study had a prospective protocol or analysis plan. If a prospective analysis plan (from your funding proposal, IRB or other ethics committee submission, study protocol, or other planning document written before analyzing the data) was used in designing the study, please include the relevant document(s) with your revised manuscript as a Supporting Information file to be published alongside your study and cite it in the Methods section. A legend for this file should be included at the end of your manuscript. If no such document exists, please make sure that the Methods section transparently describes when analyses were planned, and when/why any data-driven changes to analyses took place. Changes in the analysis, including those made in response to peer review comments, should be identified as such in the Methods section of the paper, with rationale.

**Reply**: We have added a sentence stating that we did not rely on a prospective protocol or analysis plan.
