# Supplementary material for: Associations between hematologic dynamics during pregnancy and obstetric complications: A retrospective observational study
Source: PLoS Med. 2026 May 20;23(5):e1004747. doi: 10.1371/journal.pmed.1004747 (PMC13215607; doi:10.1371/journal.pmed.1004747)
Supplement: Attachment — Submitted filename: response_to_reviewers.docx [file pmed.1004747.s025.docx]

April 8th, 2026

Dear Dr. De Bruijn,

Thank you for again considering the revision of our manuscript entitled “Associations between hematologic dynamics during pregnancy and obstetric complications: a retrospective observational study” for publication in *PLOS Medicine.* We appreciate the additional comments which have helped us strengthen the manuscript. We have also performed a careful review for clarity, typos, and accuracy of the reported values throughout.

We have provided a detailed, point-by-point response and description of the corresponding manuscript revisions in the following text.

**Comments from Reviewers**

Reviewer #1: The revisions have made the manuscript better.
The limitations would be the generalizability of this analysis to the population of pregnant people with preterm HDP requiring delivery (i.e <28w).

**Reply:** *We thank the reviewer for their comments.*

Reviewer #2: I thank the authors for their considered update of the manuscript, they have responded to all reviewers well.

**Reply:** *We thank the reviewer for their comments.*

**Editorial Requests**

(R.1) Please confirm that your title complies with PLOS Medicine's style. Your title must be nondeclarative and not a question. It should begin with main concept if possible. "Effect of" should be used only if causality can be inferred, i.e., for an RCT. Please place the study design ("A randomized controlled trial," "A retrospective study," "A modelling study," etc.) in the subtitle (ie, after a colon). Please change your title to: “Associations between hematologic dynamics during pregnancy and obstetric complications: a retrospective observational study”

**Reply:** *We have changed our title to reflect this request.*

(R.2) Please ensure that all abbreviations are defined at first use throughout the text.

**Reply:** *We have added definitions throughout the text where missing*

(R.3) Consider using the full names of some of the indices in the results, and/or the discussion, for ease of reading for non-experts. I do appreciate/acknowledge they are already defined in the methods.

**Reply:** *Thank you for the feedback; we have altered the Results and Discussion to include the full names of indices the first time they are mentioned in these sections.*

(R.4) Please confirm that all numbers presented in the abstract are present and identical to numbers presented in the main manuscript text.

**Reply:** *We have double-checked the numbers in the abstract and aligned them so that all are in the validation cohort. They match the text.*

(R.5) Competing interests: Thank you for including your competing interests. Please include a sentence "All other authors have no competing interests to declare".

**Reply:** *We have added this sentence to the conflicts of interest section.*

(R.6) Funding: Please provide URLs for all funders. Please also ensure it is clear that the T32 grant is an NIH grant.

**Reply:** *We have specified the nature of the T32 grant and added URLs for all funders.*

(R.7) Ethics statement. Thank you for including your ethics statement. 
   -Please use the full name of the MBG institutional review board, rather than the abbreviation.
   -Please ensure that the ethics statement is correctly placed/referenced. In the meta-data, it is stated that the ethics statement is included in the methods, whereas in reality it can be found after the discussion in the manuscript.

**Reply:** *We have added the full name of the MGB IRB and moved the paragraph under Methods.*

(R.8) Regarding the comment from Reviewer 4, comment 3: We think the response to this comment in your revision is adequate. However, please include a sentence in the methods, that you conducted a systematic evaluation of parameter stability.

**Reply:** *We have added a sentence as requested.*

**Methods**

**Page 5, Line 169-171**

After conducting a systematic evaluation of CBC parameter stability, we did not consider mean platelet volume due to shifts across hematological analyzers (**Figure S1**).

DATA AVAILABILITY
(R.9) PLOS Medicine requires that the de-identified data underlying the specific results in a published article be made available, without restrictions on access, in a public repository or as Supporting Information at the time of article publication, provided it is legal and ethical to do so. Please see the policy at   <http://secure-web.cisco.com/1HDDw1URo4K0kDQxxdbJTFW9th_OmbDFFKZbYpCgedyJL0ibWeg5A723WWb2kC-HtkQbghKYm4ug2RfWPqhG8D1P9vN9VCr9IUXD7FcXdg0CSkdNrnPuOehXsFDDNrprPVcEvGIE2dDG-GB5Fh4ah1wXkjmr6EUlFd_zo5P3OVYk4BtybGi-_sqMfEguvMPpH3rX7JNGrvXmR7RjNrNxYXVYcSTBeDTHOQaMKKK6qkpSgU3CzIlfrr5xHP0zJyK9VTDcg5LbM7taF3Kg67YMnYqsRbNzxp48b8qdTr1gTWlgpWIpIQnb3jqTNHntVQmwb-p1g_d1kuieqxDj_-JKp-A/http%3A%2F%2Fjournals.plos.org%2Fplosmedicine%2Fs%2Fdata-availability>   and FAQs at   <http://secure-web.cisco.com/1lFhMyFvVTYQDjb5Jqj9pFMFrgsSgqvDE7TCLt2SK5ewcKeR3sI7lTwrh0CBYbGOn3HsRdiQUSPZA7qM1JrEoU0ys-d3EySnD_7oEhMoMnWB4XO8VOnoZIRtvdD7nx8YQRxHWxsILxGpRQOghsF5CDTpeiHUQEIEI5-BSV_GPV8jHLJPb0-SEDuUvyEK2fSYHpMMKz2Uzi0fFBYb7egD56NL5wDhglodb27wPPGX52lOH6Gay2Yg8Dd_L5DNsEhdIHucqsWiu5uBWD8iFMWd_dc_yS2j3aM5Zn0P3FBM71OnA5iV3PA8yC2oxoQERTiG54xiJpGEGSQfwnofffe8C0g/http%3A%2F%2Fjournals.plos.org%2Fplosmedicine%2Fs%2Fdata-availability%23loc-faqs-for-data-policy>  
PLOS defines the “minimal data set” to consist of the data set used to reach the conclusions drawn in the manuscript with related metadata and methods, and any additional data required to replicate the reported study findings in their entirety. Authors do not need to submit their entire data set, or the raw data collected during an investigation. Please submit the following data:
-The values behind the means, standard deviations and other measures reported;
-The values used to build graphs;
-The points extracted from images for analysis."

**Reply:** *We now provide additional Supplementary Tables with the aggregated data used to produce the images.*

(R.10) Please include contact information regarding where the data can be obtained, and note this cannot be the study author.

**Reply:** *We have provided aggregated data; individual data is not available for sharing due to IRB and privacy restrictions. We have updated the text accordingly.*

(R.11) Please clarify whether the aggregated data can also not be shared legally, or include them in the manuscript as a supporting file.

**Reply:** *We now provide additional Supplementary Tables with the aggregated data used to produce the images. Specifically, we added Table S5 with percentile values to make the longitudinal dynamics panels of Figure 3. Aggregate data to plot Figure 3 are in the following tables: Table S3, S4, S5. Aggregate data to plot Figure 4 are in the following tables: Table S8, S10.*

(R.12) Figure 3: please include a statement in the legend that the numerical values can be found in table S3.

**Reply:** *We have added the requested sentence.*

ABSTRACT

(R.13) Please confirm that your abstract complies with our requirements, including format (three sections: Background, Methods and Findings, and Conclusions) and providing all the information relevant to this study type <https://secure-web.cisco.com/1qD-UZzE7SK4VwJ9DhD22cMuahyZv6nCj4BZn0YD0XIBhn1vwjvaX71dkQPD-OoZqynNqdOdlvLatMq9Kygbj9nKEl8OBMWHNLwfkTqWutaSOKTwVVQhu21iscgE5nCiiDoYvUFPzByV5-DdRLVLFlIbvtrroOfjGQ7yhpmQ_dguYW1M2CsPu3SizsaqnBo-oRK2FhmTWgk9IctS3vb_EvSEZJFFTqi9SC8-6dUmFwWgIm_ySDmfUhNyvD0iUje1uuT5r9Oc4f6d2VLWeKvEEtcCmAO3xKzMCrhiX9G1tEQqQobqiy5-r3_24jfUwfj1ocJw-WexTpX9SWzpsoGQlcA/https%3A%2F%2Fjournals.plos.org%2Fplosmedicine%2Fs%2Fsubmission-guidelines%23loc-abstract>

**Reply:** *We confirm that the abstract complies with PLOS Medicine requirements.*

(R.14) Abstract: Please consider whether any of gestational-age-specific reference intervals, specifically the ones that differ from the literature, would be worth including in the abstract. We mean the numerical values here, not just a description.

**Reply:** *We appreciate this suggestion. However, since we would have to report the ranges of 5 indices across multiple time periods, we have opted not to include them in the abstract for ease of reading.*

(R.15) Please mention the specific data source (e.g. : EHR-based Maternal Health Cohort; Massachusetts General Hospital) rather than the current description "from electronic health records at an academic medical center and affiliated health system in the United States".  If this is not possible, please provide an explanation why.

**Reply:** *We have changed our abstract to specify the data sources we developed our discovery and validation cohorts from, as requested.*

(R.16) In the last sentence of the Abstract Methods and Findings section, please describe the main limitation(s) of the study's methodology.

**Reply:** *We have now added a sentence.*

AUTHOR SUMMARY
(R.17) In the author summary, in the final bullet point of 'What Do These Findings Mean?', please include the main limitations of the study in non-technical language.

**Reply:** *We have now added a sentence.*


FIGURES AND TABLES
(R.18) Please consider avoiding the use of red and green in order to make your figure more accessible. This is for Figure 1, Figure 3, as well as figures S3 and S7.

**Reply:** *We have updated the figures to remove the use of green, as suggested.*

(R.19) When a p value is given, please specify the statistical test used to determine it in the legend. Please check this for all figures and tables, but specifically table S2, S4, S6-12.

**Reply:** *We have updated all figure and table legends to report the statistical tests used to generate p-values.*

(R.20) Please provide titles and legends for all figures and tables (including those in Supporting Information files).  It is currently not completely clear what is the title, and what is the legend for each figure/table.

**Reply:** *We have now bolded the titles for all figures and tables.*


**Technical requests**

1) Please note that the contact for accessing your data cannot be an author on the manuscript. Please therefore replace the current contact information with a third party contact, such as an institutional data access committee.

**Reply:** *We have provided aggregated data; individual data is not available for sharing due to IRB and privacy restrictions. Given the inability to share the data, we have not provided an institutional contact.*
